# Revisiting the global workspace orchestrating the hierarchical organization of the human brain

**DOI:** 10.1038/s41562-020-01003-6

**Published:** 2021-01-04

**Authors:** Gustavo Deco, Diego Vidaurre, Morten L. Kringelbach

**Affiliations:** 1 Center for Brain and Cognition, Computational Neuroscience Group, Department of Information and Communication Technologies, Universitat Pompeu Fabra, Barcelona, Spain; 2 Institució Catalana de la Recerca i Estudis Avançats (ICREA), Barcelona, Spain; 3 Department of Neuropsychology, Max Planck Institute for Human Cognitive and Brain Sciences, Leipzig, Germany; 4 School of Psychological Sciences, Monash University, Clayton, Victoria Australia; 5 Department of Psychiatry, University of Oxford, Oxford, UK; 6 Center for Functionally Integrative Neuroscience, Department of Clinical Medicine, Aarhus University, Aarhus, Denmark; 7 Centre for Eudaimonia and Human Flourishing, University of Oxford, Oxford, UK; 8 Center for Music in the Brain, Department of Clinical Medicine, Aarhus University, Aarhus, Denmark

**Keywords:** Neuroscience, Psychology, Cognitive control

## Abstract

A central challenge in neuroscience is how the brain organizes the information necessary to orchestrate behaviour. Arguably, this whole-brain orchestration is carried out by a core subset of integrative brain regions, a ‘global workspace’, but its constitutive regions remain unclear. We quantified the global workspace as the common regions across seven tasks as well as rest, in a common ‘functional rich club’. To identify this functional rich club, we determined the information flow between brain regions by means of a normalized directed transfer entropy framework applied to multimodal neuroimaging data from 1,003 healthy participants and validated in participants with retest data. This revealed a set of regions orchestrating information from perceptual, long-term memory, evaluative and attentional systems. We confirmed the causal significance and robustness of our results by systematically lesioning a generative whole-brain model. Overall, this framework describes a complex choreography of the functional hierarchical organization of the human brain.

## Abstract (web summary)

Deco et al. use multimodal neuroimaging data to quantify the global workspace as the common ‘functional rich club’ of regions intersecting across seven tasks as well as rest.

## Main

Over recent decades, research within systems neuroscience has suggested that the brain is hierarchically organized in terms of anatomical structure^1–7^ and function^8–12^. Nevertheless, a key remaining challenge is to determine precisely how this hierarchical organization allows the brain to orchestrate function and behaviour by organizing the flow of information and the underlying computations necessary for survival.

A large body of research has argued that whole-brain orchestration is likely to be carried out by a core subset of integrative brain regions. For example, according to the classic model of Norman and Shallice^13^, this processing involves the prefrontal cortices in charge of the supervisory attentional regulation of lower-level sensorimotor chains. In contrast, Baars proposed the concept of a ‘global workspace’ (GW), where information is integrated in a small group of brain regions before being broadcast to many other regions across the whole brain^14^. Extending this framework, Dehaene and Changeux^15^ proposed their ‘global neuronal workspace’ hypothesis that associative perceptual, motor, attention, memory and value areas interconnect to form a higher-level unified space where information is broadly shared and broadcast back to lower-level processors. Colloquially, the GW is thus akin to a small core assembly of people in charge of an organization. Larger brain network organization has been shown to be efficient, robust and largely fault tolerant^4,16,17^, yet the effects of lesioning a core assembly—like the GW—are currently unknown.

Until recently, a key obstacle to advancing our understanding of the human brain’s functional hierarchical organization has been the lack of suitable whole-brain measurements. However, the advent of Big Data, such as the Human Connectome Project (HCP)^18,19^, has created large multimodal whole-brain neuroimaging datasets of healthy individuals both in resting state and whilst performing many different tasks. The development of more advanced neuroimaging methods could allow for the estimation of the bi-directional flow of information between all regions across the whole brain, which could subsequently be used to characterize the functional hierarchical organization of the brain. Many methods exist to estimate the information flow using different neuroimaging modalities, which have greatly contributed to our current knowledge (for example, refs. ^20–22^). Here, we use this method to address the key challenge of identifying the functional hierarchical organization of the brain.

The main aim of our study is to characterize the GW as the core set of brain regions responsible for integration and orchestration. To do this, we have to determine the functional hierarchical whole-brain organization. For this purpose, we propose the concept of a ‘functional rich club’ (FRIC) as the core set of regions, an array of functional hubs that are characterized by a tendency to be more densely functionally connected among themselves than to other brain regions from where they receive integrative information. This notion is related to previous static descriptions of the anatomical rich club^5,7^, which includes nodes in a network with a tendency for high-degree nodes to be more densely connected among themselves than nodes of a lower degree. However, unlike the anatomical rich club, FRIC is a dynamic measure based on bidirectional flow of information not constrained by anatomy and thus will change across different tasks. Using our colloquial example of a core assembly, for different tasks some people remain through all executive meetings while others are substituted in and out on the basis of their expertise. In a similar manner, FRIC would include both common and task-specific brain regions as a result of the different flows of information for different kinds of task. Following the original ideas of Baars^14^, we propose that the invariant ‘GW’ is the intersection of the different sets of task-related FRICs.

To achieve our goal of identifying the GW as the intersection of FRICs from data of different tasks and in resting condition, we develop a normalized directed transfer entropy (NDTE) framework. This framework provides a bidirectional description of the functional information flow underlying brain signals from 1,003 HCP participants in resting state and when carrying out seven tasks.

Finally, we validate our finding of the invariant GW through constructing and selectively lesioning a whole-brain model that accurately simulates the empirical functional hierarchy and thus describes the underlying dynamical mechanisms. Systematic lesioning of subsets of regions in the FRIC in this model establishes the generative role of the GW in orchestrating function and allows us to characterize its efficiency and robustness.

## Results

**Fig. 1:**
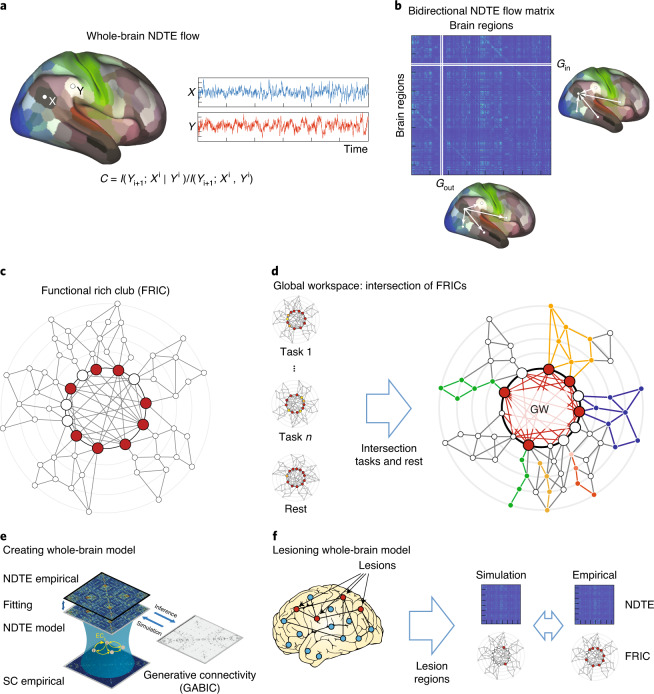
Overview of general theoretical framework. **a**, The causal bidirectional flow of information between any two brain regions is determined by computing the pairwise NDTE. The figure shows two arbitrary regions, X and Y, superimposed on the human brain, with different shades of colours marking different regions, and we show the timecourses of X and Y in blue and red, respectively. The statistical significance is determined at the individual level using the circular time-shifted surrogates method^23^ and at the group level using *P* level aggregation across individuals. **b**, The functional hierarchical organization is given by the full NDTE matrix, where the rows contain the target regions and the columns contain the source regions. For each brain region, the total incoming flow of information, *G*_in_, is simply the sum of all sources (that is, the sum over the rows in the matrix). Similarly, the total outgoing flow of information, *G*_out_, is the sum over all targets (that is, the sum over the columns). **c**, The FRIC is the smallest set of brain regions (highlighed in red) that integrate and orchestrate function in a given task. It can be identified as the most highly connected brain regions that (1) are more densely connected within themselves than to regions with lower connectivity, whilst (2) having the highest level of incoming directed flow (*G*_in_) and (3) the lowest outgoing directed flow (*G*_out_; see Methods). **d**, The global neuronal workspace must be relevant to all tasks and situations (highlighted with red circles for each task) and must therefore comprise the common FRIC members across many different tasks, that is, the intersection of FRICs from tasks and rest (highlighted with red circles on the right panel, which also shows networks lower in the hierarchy in different colours). **e**, To establish the causal importance of the FRIC, we fit a whole-brain model to the resting NDTE empirical data and extract the underlying effective connectivity (see Results and Methods). **f**, The whole-brain model is then systematically lesioned for regions belonging to the FRIC and compared with lesioning non-FRIC members. For illustration, the cartoon highlights the lesion sites with red dots and other regions with blue dots. Overall, this confirms the causal importance of these regions in the orchestration of the functional hierarchical organization of the human brain.

Figure 1 sketches the overall aim of finding the regions orchestrating the functional hierarchical organization of the brain, sometimes called the ‘global workspace’^14,15^.

### Functional hierarchical organization of resting

We characterized the functional hierarchical organization of the resting state of 1,003 participants. For this purpose, we extracted the bidirectional flow of information between brain regions using the concept of NDTE (see Methods), which is an information-theoretical measure of causality between two time series. This allows us to infer the underlying bidirectional reciprocal communication between any source and target regions. Specifically, first we compute the mutual information directed flow, that is the predictability of a target in the future given the past of the source region, beyond the predictability from its own past (see Eq. 1 in Methods). Then the mutual information directed flow is normalized by the mutual information that both source and target have about the future of the target (see Eq. 8 in Methods). At the individual level, we compute the statistical significance by using the circular time-shifted surrogates method, which has been shown to be particularly well adapted to causal measurements^23^. At the group level, we aggregate the *P* values corresponding to each pairwise NDTE flow using the Stouffer method^24^ and correct for multiple comparisons (see Methods).

Importantly, the NDTE framework was thoroughly validated through network simulations (see Methods and Supplementary materials). Furthermore, in the Methods and Discussion, we show how other research has validated the transfer entropy framework in neuroimaging^25^ and the relevance of using circular shift surrogates for eliminating spurious inferences^26,27^.

We computed a matrix containing the NDTE flow between the regions in four different parcellations (see Methods). For a fine-scale parcellation, we used a modified version of the Glasser parcellation with a total of 378 regions (360 cortical and 18 subcortical regions)^28^. For a medium-scale parcellation, we used the Brainnetome parcellation with a total of 246 regions (210 cortical and 36 subcortical regions)^29^. For a coarser-scale parcellation suitable for whole-brain computational modelling, we used a modified Desikan–Killiany parcellation which included subcortical regions (DK80, 62 cortical regions and 18 subcortical regions)^30,31^.

To establish the hierarchical organization, we compute the total incoming and outgoing information for all brain regions. More specifically, for each brain region, the total incoming flow of information, *G*_in_, is the sum of all sources (that is, the sum over the rows in the matrix). Similarly, the total outgoing flow of information, *G*_out_, is the sum over all targets (that is, the sum over the columns). We also compute the total information being processed in a brain region as the sum: *G*_tot_ = *G*_in_ + *G*_out_, which is possible given that the measures have been normalized (see above and Methods).

**Fig. 2:**
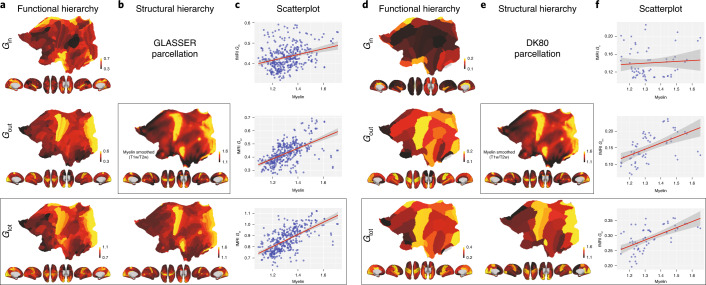
Comparing functional and structural hierarchical organization. **a**, Functional hierarchy shown as cortical renderings of each of the incoming (*G*_in_), outgoing (*G*_out_) and total (*G*_tot_) directional information flow computed from the NDTE matrix of 1,003 HCP participants (see Fig. 1 and Methods) in the Glasser parcellation (using renderings of cortical flattening and 3D renderings with midline, right, left, top and bottom views). As shown, the outgoing information, *G*_out_, is highest in sensory areas, while the incoming information, *G*_in_, is highest in higher-order, integrative transmodal areas. **b**, The structural hierarchy shown for myelination of brain regions (myelin-weighted T1w/T2w) with the same renderings at the voxel level (top) and in the Glasser parcellation (bottom). **c**, Scatterplots between the functional hierarchy (*G*_in_, *G*_out_ and *G*_tot_) and structural hierarchy (myelination). The linear correlations are shown by the red line (with standard error in shaded gray) overlaid on the scatterplots (see Results for *r* values). This shows that myelination is highly correlated with *G*_tot_ and mainly driven by correlation with *G*_out_. On the other hand, there is a much lower correlation with the incoming flow, that is, an integrative measure of *G*_in_. This means that the static measure of myelination is likely to mostly reflect the driving flow in sensory areas, but provides much less information on integrative areas. **d**, The same as **a** but for the DK80 parcellation. **e**, Myelination in the DK80 parcellation. **f**, Scatterplots between the functional hierarchy (*G*_in_, *G*_out_ and *G*_tot_) and structural hierarchy (myelination) in the DK80 parcellation. The linear correlations are shown by the red line (with standard error in shaded gray) overlaid on the scatterplots (see Results for *r* values). The results in this coarser-scale DK80 parcellation are fully consistent with the finer-scale Glasser parcellation.

**Extended Data Fig. 1:**
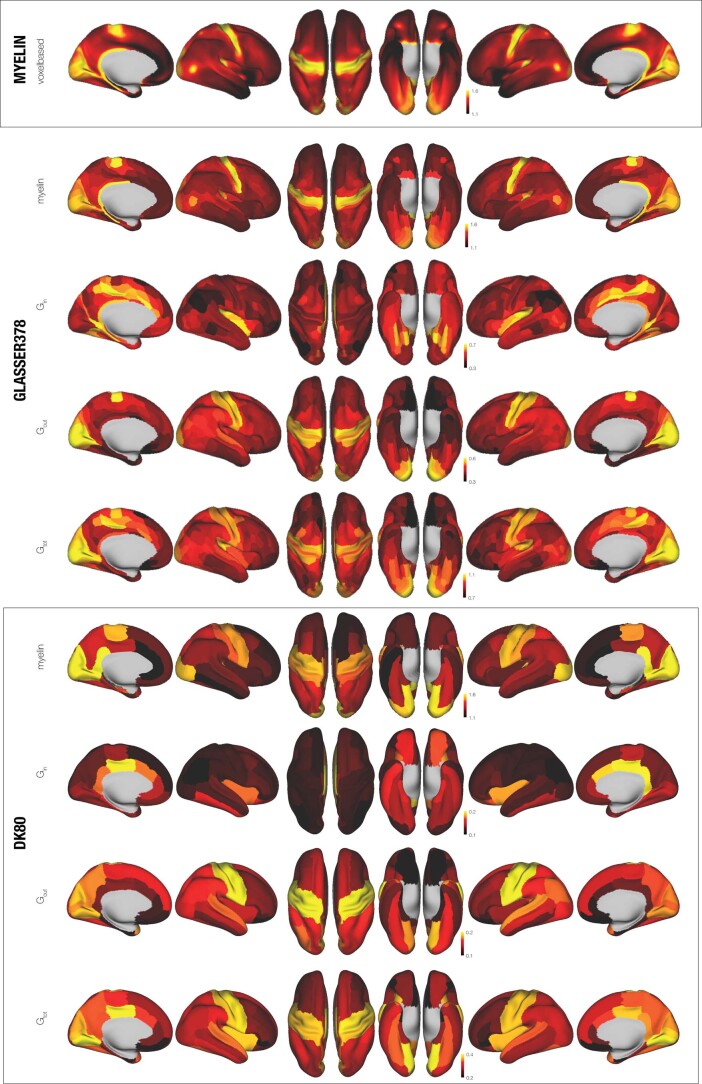
Functional hierarchy of resting state found using NDTE rendered on Glasser and DK80 parcellations. At the top is shown the full cortical voxelbased renderings of myelin (T1w/T2w). Below is shown renderings for both the Glasser and DK80 parcellations of myelin and the NDTE measures (G_in_, G_out_ and G_tot_) in 1003 HCP participants.

We show the functional hierarchy described by each of the incoming (*G*_in_), outgoing (*G*_out_) and total (*G*_tot_) directional information flow computed from the NDTE matrix of 1,003 participants (see Fig. 1 and Methods) in the Glasser (Fig. 2a) and DK80 parcellations (Fig. 2d). As can be seen (Extended Data Fig. 1), the outgoing information flow, *G*_out_, is highest in sensory areas, while the incoming information, *G*_in_, is highest in higher-order, integrative transmodal areas.

On the other hand, a popular proxy for anatomical hierarchy is the myelination of brain regions as measured by myelin-weighted T1w/T2w^32^. It should be noted that there is controversy in the literature as to whether the ratio of T1w and T2w images (T1w/T2w ratio) in grey matter is a reliable estimate of cortical myelin content. Nevertheless, there is a growing consensus that the T1w/T2w ratio is a marker of general white-matter microstructure in both health and disease^33–35^ provided that a correctly standardized measure of T1w/T2w is applied^36^. Our analysis uses the neuroimaging in the HCP dataset of healthy participants with isotropic spatial resolution of 0.7 mm, which enables highly accurate individual maps of cortical myelin content and thickness^18^.

We render the T1w/T2w in the Glasser (Fig. 2b and Supplementary Fig. 1) and DK80 parcellations (Fig. 2e and Supplementary Fig. 1). Important information about the driving nature of sensory areas (more myelin) and the integrative role of higher-order transmodal areas (less myelin) has been demonstrated from this structural information in recent papers^8,37,38^. However, this structural measure does not change with different tasks and is therefore unlikely to capture the functional dynamic changes in hierarchical organization.

We provide scatterplots between the functional hierarchy (*G*_in_, *G*_out_ and *G*_tot_) and structural hierarchy (myelination) for the cortical regions in the Glasser (Fig. 2c) and DK80 parcellations (Fig. 2f). The linear Pearson correlations are shown by the red line (with standard error in shaded gray) overlaid on the scatterplots, with values for the Glasser pacellation of *r*(720) = 0.29 for *G*_in_ (*P* < 0.001, 95% CI 0.19–0.38), *r*(720) = 0.57 for *G*_out_ (*P* < 0.001, 95% CI = 0.50–0.64) and *r*(720) = 0.63 for *G*_tot_ (*P* < 0.001, 95% CI = 0.56–0.69) and values for the DK80 parcellation of *r*(124) = 0.07 for *G*_in_ (not significant, 95% CI −0.18 to 0.32), *r*(124) = 0.53 for *G*_out_ (*P* < 0.001, 95% CI 0.32–0.69) and *r*(124) = 0.61 for *G*_tot_ (*P* < 0.001, 95% CI 0.42–0.74). This shows that myelination is highly correlated with *G*_tot_, and mainly driven by correlation with the outgoing flow, *G*_out_. Importantly, as expected, the level of correlation between function and structure is lower for the incoming flow (the integrative measure of *G*_in_). This is consistent with the demonstration of a direct link between electrophysiological connectivity and myelination^39^.

**Extended Data Fig. 2:**
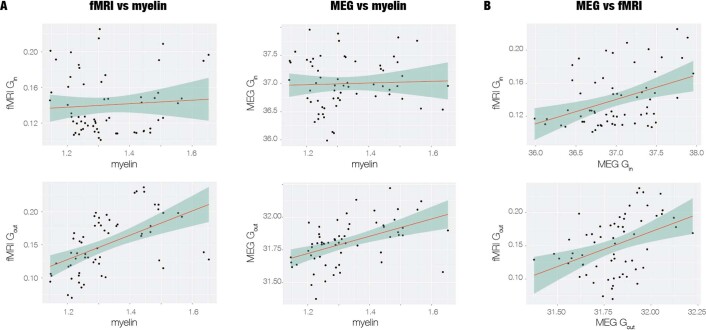
Validating NDTE framework using MEG from 89 HCP participants. We used the NDTE framework on MEG resting state data from 89 HCP participants (each having three resting state sessions) and extracted the timeseries from the 62 cortical regions of the DK80 parcellation (see Methods). On the left is shown the scatterplots for G_in_ and G_out_ for fMRI resting state data versus myelin (T1w/T2w). On the right is shown the same scatterplots for G_in_ and G_out_ for MEG resting state data versus myelin. The Pearson correlation values are r(124)=0.04 for G_in_ (14-22 Hz, window size 1000 ms, not significant, 95% CI = -0.21–0.29), r(124)=0.48 for G_out_ (22.5-30.5 Hz, window size 500 ms, P < 0.001, 95% CI = 0.26–0.65). B) The figure shows the scatterplots between G_in_ and G_out_ for MEG and fMRI data with correlation values of r(124)=0.40 for G_in_ (P < 0.001, 95% CI = 0.17–0.59) and r(124)=0.41 for G_out_ (P < 0.001, 95% CI = 0.18–0.60).

To validate the results from functional magnetic resonance imaging (fMRI), we also characterized the functional hierarchical organization for the corresponding HCP magnetoencephalography (MEG) time series in the 62 cortical regions of the DK80 parcellation. Attesting to the robustness of the results, we found similar correlations between *G*_in_ and *G*_out_ from HCP MEG data and fMRI: *r*(124) = 0.40 for *G*_in_ (14–22 Hz, window size 1,000 ms, *P* < 0.001, 95% CI 0.17–0.59), *r*(124) = 0.41 for *G*_out_ (22.5–30.5 Hz, window size 500 ms, *P* < 0.001, 95% CI 0.18–0.60) (see Extended Data Fig. 2b). HCP MEG data and T1w/T2w: *r*(124) = 0.04 for *G*_in_ (14–22 Hz, window size 1,000 ms, not significant, 95% CI −0.21 to 0.29), *r*(124) = 0.48 for *G*_out_ (22.5–30.5 Hz, window size 500 ms, *P* < 0.001, 95% CI 0.26–0.65) (see Extended Data Fig. 2a).

### Functional hierarchy across different tasks and rest

These results show that the measures of *G*_in_ and *G*_out_ are very different, and we quantified the changes in the relationship between the functional hierarchy across different tasks and rest. We used all seven tasks in the HCP dataset. The task battery was designed to cover as wide a range of neural systems as is feasible within realistic time constraints^40^ (see Methods).

**Fig. 3:**
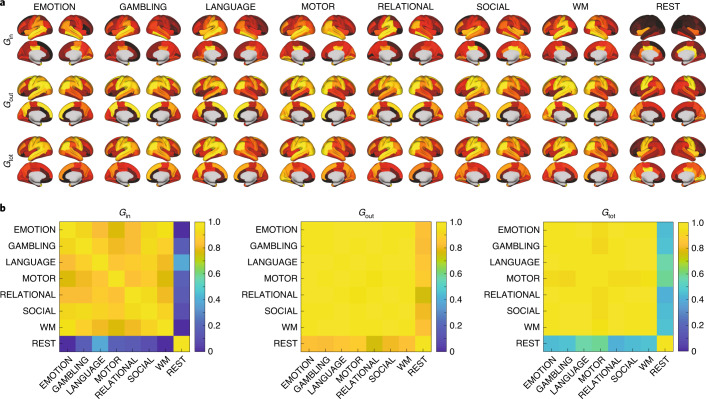
Functional hierarchy across different tasks and rest. **a**, Cortical renderings of all seven tasks and rest for the incoming (*G*_in_), outgoing (*G*_out_) and total (*G*_tot_) directional information flow computed from the NDTE matrix of 1,003 HCP participants (see Fig. 1 and Methods) in the DK80 parcellation (using 3D views from the side and midline). **b**, Matrices of the comparison of incoming (*G*_in_), outgoing (*G*_out_) and total (*G*_tot_) directional information flows in the seven tasks and rest. As can be clearly seen, the *G*_in_ matrix and the renderings of the incoming flows of information (receivers) are significantly different between tasks and rest. This suggests that different tasks process incoming flows differently. This is in contrast to the *G*_out_ matrix and the renderings of the outgoing flows of information (drivers), which are similar. This shows that sensory areas are consistently driving the information flow. Interestingly, as can be seen from the *G*_tot_ matrix, the total processing of information flow is more similar within the seven tasks than compared with rest, suggesting the extrinsic, sensory nature of task processing compared with the intrinsic nature of resting-state processing.

**Extended Data Fig. 3:**
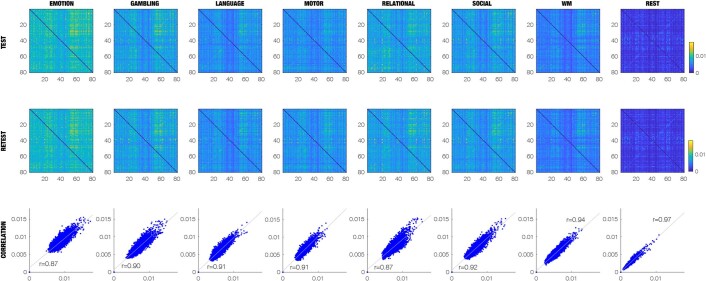
Validation of NDTE framework on test-retest data for task and rest. We validated the NDTE framework on test-retest data and found very high reproducibility (of up to a 0.97 correlation) using only a subsample of 45 participants of the 1003 participants. The correlation values for the rest-retest are the following: EMOTION: r(3160)=0.866, P < 0.001, 95% CI = 0.860–0.872); GAMBLING: r(3160)=0.898, P < 0.001, 95% CI = 0.893–0.903); LANGUAGE: r(3160)=0.907, P < 0.001, 95% CI = 0.902–0.911); MOTOR: r(3160)=0.907, P < 0.001, 95% CI = 0.903–0.912); RELATIONAL: r(3160)=0.866, P < 0.001, 95% CI = 0.860–0.872); SOCIAL: r(3160)=0.924, P < 0.001, 95% CI = 0.920–0.927); WM r(3160)=0.939, P < 0.001, 95% CI = 0.936–0.942); REST: r(3160)=0.970, P < 0.001, 95% CI = 0.968–0.971).

Figure 3a provides cortical renderings of all seven tasks and rest for the incoming (*G*_in_), outgoing (*G*_out_) and total (*G*_tot_) directional information flow computed from the NDTE matrix of participants (see Fig. 1 and Methods) in the DK80 parcellation (using 3D views from the side and midline). Importantly, we validated the NDTE framework on 45 HCP participants with retest data (Extended Data Fig. 3) and found very high reproducibility (correlation up to a 0.97) despite using only a subsample of 45 of the 1,003 participants.

**Extended Data Fig. 4:**
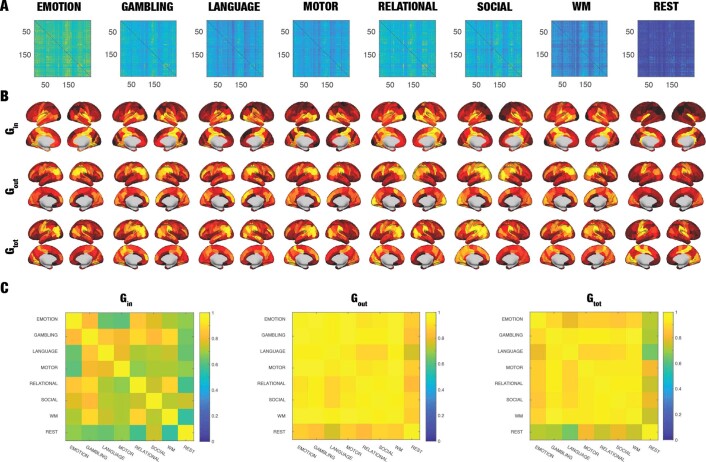
Functional hierarchy across different tasks and rest in Brainnetome246 parcellation. **a**, NDTE matrices for all seven tasks and resting state from all HCP participants in the BN246 parcellation. **b**, Cortical renderings of the incoming (G_in_), outgoing (G_out_) and total (G_tot_) directional information flow computed from the NDTE matrix using 3D views from the side and midline (similar to Fig. 3). **c**, Matrices of the comparison of incoming (G_in_), outgoing (G_out_) and total (G_tot_) directional information flow in the seven tasks and rest. Similar to the results for the DK80 and Glasser378 parcellations, the renderings of the incoming flow of information (receivers) are significantly different between tasks and rest.

We also computed and rendered the NDTE matrices in the finer Brainnetome246 parcellation for all seven tasks and rest for the incoming (*G*_in_), outgoing (*G*_out_) and total (*G*_tot_) directional information flow for all participants (see Extended Data Fig. 4 and Methods). The results are very similar and consistent across tasks and rest in the two parcellations.

Quantification of the differences is provided by the correlation matrices between incoming (*G*_in_), outgoing (*G*_out_) and total (*G*_tot_) directional information flow in the seven tasks and rest. The results show that there are significant differences between the hierarchy in tasks and rest for *G*_in_ (receivers), which reflects that different tasks process incoming information differently. On the other hand, outgoing flow of information (drivers) is similar across tasks, suggesting that sensory areas are consistently driving the information flow. Importantly, as shown in the *G*_tot_ matrix, the total processing of information flow is more similar within the seven tasks than compared with rest.

### Quantifying the FRIC in tasks and resting state

In the structural domain, Zamora-Lopez, Van Heuvel and Sporns proposed the concept of a structural ‘rich club’^5,7^, which is characterized by a tendency for high-degree brain regions to be more densely connected among themselves than regions of a lower degree, providing important information on the higher-level topology of the brain network. We extend this concept to the functional domain by defining the concept of a FRIC, which crucially is not static but changes between tasks and rest (see Methods).

**Fig. 4:**
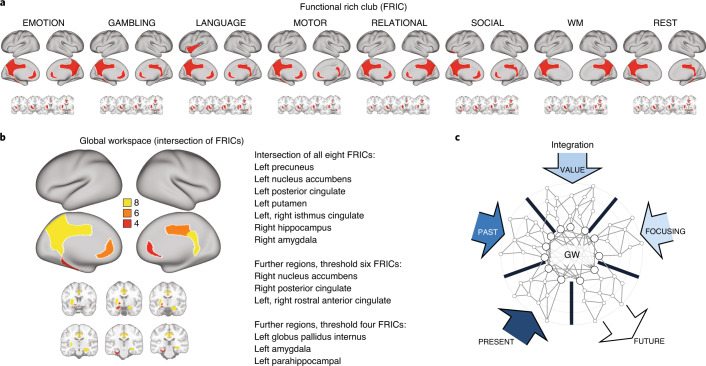
Identifying the GW as the intersection of FRICs for rest and seven tasks. **a**, We computed the functional hierarchical organization of all seven tasks (emotion, gambling, language, motor, relational, social and working memory) and rest for the HCP participants. This allows us to compute the FRIC as the set of regions that define a ‘club’ of functional hubs characterized by a tendency to be more densely functionally connected among themselves than to other brain regions from where they receive integrative information (see Methods). As can be seen, these FRICs are similar but not identical across tasks and rest. **b**, We compute the regions in the GW as the intersection of the FRIC members across all possible tasks and resting state. Here, we used the maximal amount of tasks available to provide a reliable estimate of the GW. At the bottom of the figure, we show a rendering of the cortical and subcortical regions in the GW. The FRIC regions for all seven tasks and rest defining the GW are the following eight brain regions: left precuneus, left nucleus accumbens, left putamen, left posterior cingulate cortex, right hippocampus, right amygdala and left and right isthmus cingulate. Lowering the threshold for participation to more than six FRICs adds two further regions: right nucleus accumbens and right posterior cingulate (in seven FRICs) and left and right rostral anterior cingulate (in six FRICs). Further lowering the threshold to four FRICs includes another three brain regions: left amygdala and left globus pallidus internus (in five FRICs) and left parahippocampal cortex (in four FRICs). **c**, These regions fit well with the idea suggested by Dehaene and Changeux that the GW is ideally placed for integrating information from perceptual (PRESENT), long-term memory (PAST), evaluative (VALUE) and attentional (FOCUSING) systems.

We computed the NDTE matrices for the DK80 parcellation for all seven tasks and resting state. This allows us to compute the FRIC as the set of regions that define a ‘club’ of functional hubs characterized by a tendency to be more densely functionally connected among themselves than to other brain regions from where they receive integrative information (see Methods). Figure 4a shows that the FRICs across tasks and rest contain similar but not identical core regions.

### Quantifying the GW

We quantified the GW, which we defined as the intersection of FRIC members across all possible tasks and resting state. Figure 4b plots a rendering of the cortical and subcortical regions in the GW.

We found that the intersecting FRIC members for all seven tasks and rest include the following eight brain regions: left precuneus, left nucleus accumbens, left putamen, left posterior cingulate cortex, right hippocampus, right amygdala and left and right isthmus cingulate. Searching for a less restrictive definition, we further lowered the threshold to include areas only common to seven FRICs, which adds two further regions: right nucleus accumbens and right posterior cingulate, while lowering to six FRICs adds left and right rostral anterior cingulate. Moreover, we found that lowering the threshold to five FRICs added the left amygdala and left globus pallidus internus, while lowering to four FRICs added the left parahippocampal cortex. The results point to a stable core of brain regions necessary in the GW.

**Extended Data Fig. 5:**
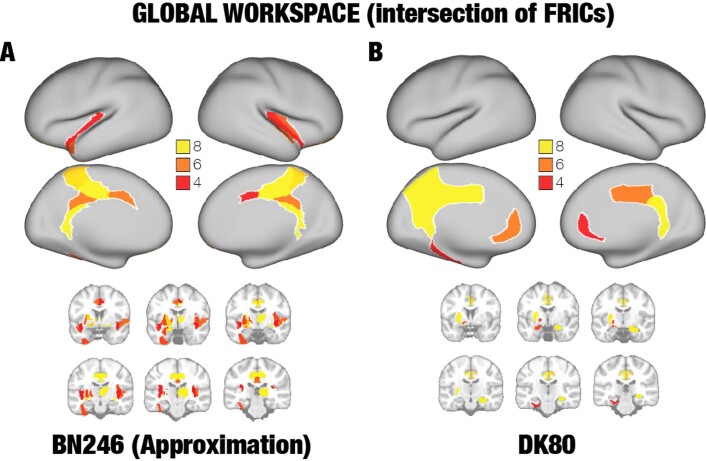
Approximation of the Global Workspace in the fine-scale Brainnetome246 parcellation for comparison with formal definition of the Global Workspace in the DK80 parcellation. **a**, The computational complexity of the FRIC algorithm makes it inappropriate for finer-scale parcellations so we developed an approximation, computing the intersection of the top G_in_ regions across rest and seven tasks. This is only an approximation since it does not conform fully to our definition of the global workspace, separate from the rest of the brain, which similar to a ‘club’ of functional hubs are characterized by a tendency to be more densely functionally connected among themselves than to other brain regions from where they receive integrative information. **b**, Nevertheless, this rough approximation shows a remarkable overlap with the Global Workspace derived from our formal definition in the FRIC algorithm applied to the DK80 parcellation (see Results and Fig. 4).

In addition to computing the GW using our formal definition, we were also prompted by a peer reviewer to investigate how this might look in a finer parcellation, the Brainnetome246 (see Methods). Unfortunately, for reasons of computational complexity, it is not possible to use our formal definition on this finer parcellation. We therefore built a method to approximate the GW as the intersection of the regions with the highest incoming information flow across tasks and rest (see Methods). Extended Data Fig. 5 shows an overlap between this rough approximation in Brainnetome246 and the fully computed GW (as the intersection of FRICs in tasks and rest) in DK80.

### Comparison with the anatomical rich club

**Extended Data Fig. 6:**
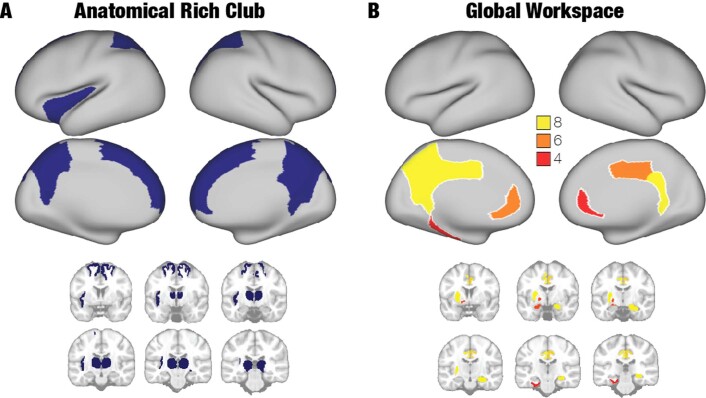
Comparing the anatomical rich club with the functional rich club (FRIC) for tasks and their intersection (Global Workspace). **a**, Using the rich club algorithm, we computed the anatomical rich club on the structural connectivity matrix for DK80. The figure shows a rendering of the ten brain regions in the rich club: left and right precuneus, left and right thalamus, left and right superior frontal, left superior parietal, left insula and right isthmus cingulate cortices. **b**, A comparison with the global workspace (shown here as well in Fig. 4 and described in details in the results) shows that they overlap in the left precuneus and the right isthmus cingulate cortex. Given that the anatomical rich club cannot change over time with functional demands, the lack of further overlap is not surprising.

The proposed FRIC concept is functional in nature, thus changing with task and capturing the top regions involved in the relevant functional organization of the task. This is different from the existing anatomical rich club concept^5,7^, which is static in nature. Extended Data Fig. 6 shows the result of computing the anatomical rich club for the structural DK80 connectivity matrix and the comparison with the FRICs of the seven tasks and their intersection in the GW. The results show that there are ten brain regions in the anatomical rich club: left and right precuneus, left and right thalamus, left and right superior frontal, left superior parietal, left insula and right isthmus cingulate cortices. Only two regions are shared with the GW, namely the left precuneus and the right isthmus cingulate cortex. Equally, the FRICs of the individual tasks and rest show a similar overlap. This demonstrates the relevance of both concepts but highlights the dynamic nature of the FRIC concept, enabling spatiotemporal characterization of the functional hierarchical organization of the brain.

### Establishing the mechanistic significance of FRIC by lesioning a whole-brain model

**Fig. 5:**
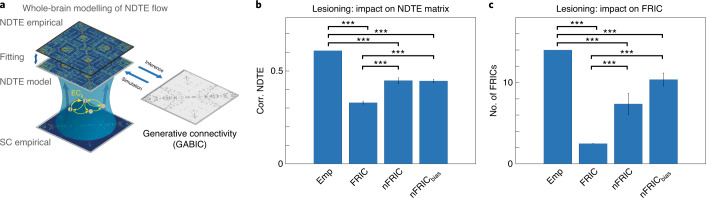
Establishing causal significance by lesioning whole-brain model. **a**, To establish the causal mechanistic functional relevance of the FRIC brain regions orchestrating the functional hierarchical organization of the resting state, we created a whole-brain model to fit the empirical NDTE flow matrix. **b**, Lesioning all FRIC compared with non-FRIC members in this whole-brain model causes a significant breakdown in information flow (see Results for significance testing). The correlation between empirical (Emp) and simulated NDTE matrices is maximally affected when FRIC members are lesioned compared both with non-FRIC members that were randomly selected (nFRIC) and with non-FRIC members that were selected using a biased method in which we chose the regions with the top 50% *G*_in_ values (nFRIC_bias_). We also compared FRIC versus nFRIC and FRIC versus nFRIC_bias_. **c**, Similarly, we found significant differences in the number of FRIC members detected when lesioning the original empirical FRIC members compared with empirical non-FRIC members, Emp versus nFRIC and Emp versus nFRIC_bias_. We also compared FRIC versus nFRIC and FRIC versus nFRIC_bias_. This is mechanistic evidence for the significance of FRIC in orchestrating functional organization. Emp, empirical; FRIC, functional rich club; nFRIC, nFRIC_bias_, non-functional rich club members.

**Extended Data Fig. 7:**
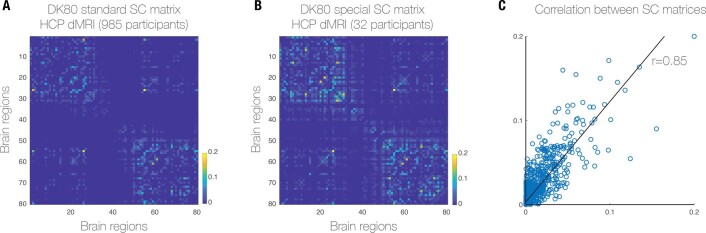
Estimation of structural connectivity (SC) matrices for DK80 using standard and special HCP datasets. **a**, The ‘standard SC matrix’ was extract from the standard HCP multi-shell dMRI data from 985 HCP participants using state-of-the-art tractography methods (see Methods). **b**, The ‘special SC matrix’ was estimated using an even better state-of-the-art multi-shell dMRI protocol taking 89 minutes for each of 32 HCP participants at the MGH centre. Please note the increased number of inter-hemispheric connections (upper right and lower left quadrants of the special SC matrix compared to the standard SC matrix). **c**, The two SC matrices are highly correlated as shown in the scatterplot (r(3160)=0.85, P < 0.001, 95% CI = 0.84–0.86).

To establish the mechanistic functional relevance of the FRIC brain regions orchestrating the functional hierarchical organization of the resting state, we derived a causal quantification from simulation. We created a whole-brain model that can generate the effective connectivity underlying the functional hierarchy (see Methods). Figure 5a shows the general framework of how this model uses optimized anatomically constrained parameters (generative anatomically constrained bidirectional connectivity (GABIC)) to describe the effective strength of the synaptic coupling to fit the NDTE matrix by maximizing the fit of the empirical and simulated NDTE matrices. The NDTE matrix is a measure of the effective connectivity in the brain, and consequently GABIC is a matrix generating this effective connectivity rather than the effective connectivity per se. The Hopf model was constructed using both the standard and special structural connectivity (SC) matrices (see Methods), where the standard matrix is estimated on 985 HCP participants while the special SC matrix is estimated using a higher-quality diffusion magnetic resonance imaging (dMRI) protocol but on only 32 HCP participants. Extended Data Fig. 7 shows that they are highly correlated (r(3,160) = 0.85, *P* < 0.001, 95% CI 0.84–0.86) but that there are slightly more interhemispheric connections in the special SC matrix compared with the standard SC matrix, suggestive of a better estimation of the true SC.

Optimizing the Hopf models using a particle swarm optimizer (see Methods) was computationally very demanding, ultimately yielding a fit with a correlation between empirical and model-generated NDTE matrices of 0.6 for both the standard and special SC matrix. Furthermore, the model NDTEs produced with both SC matrices were very highly correlated. Note that the NDTE matrix is bidirectional, thus the GABIC matrix is also asymmetric. We found no statistically significant evidence that the GABIC matrix correlated with the NDTE matrix (correlation r(3,160) = 0.0036, not significant, 95% CI 0.002–0.0047). NDTE correlated *r*(3,160) = 0.51, *P* < 0.001, 95% CI 0.511–0.513) with static functional connectivity, demonstrating that there is complementary information in the NDTE, thus further constraining the whole-brain modelling.

Figure 5b shows the result of lesioning all FRIC compared with non-FRIC members in the whole-brain model, which causes a significant breakdown in information flow. When measuring this in terms of the correlation between the empirical and simulated NDTE matrices, the relevant comparisons revealed a highly significant change in the fitting of the model when FRIC members are lesioned compared with the lesioning of non-FRIC members selected from all other regions. Specifically, we ran Wilcoxon rank-sum comparisons of the different conditions. First, we found that the correlation between the empirical (Emp) and simulated NDTE matrices is maximally affected when FRIC members are lesioned (*P* < 0.001, median difference: 0.28, 95% CI 0.27–0.29, compare first and second bars in Fig. 5b) compared both with non-FRIC members that were randomly selected (nFRIC) (*P* < 0.001, median difference 0.16, 95% CI 0.13–0.19, compare first and third bars in Fig. 5b) and with non-FRIC members that were selected using a biased method where we chose the regions with the top 50% *G*_in_ values (nFRIC_bias_) (*P* < 0.001, median difference 0.16, 95% CI 0.15–0.18, compare first and fourth bars in Fig. 5b). We also compared FRIC versus nFRIC (*P* < 0.001, median difference 0.12, 95% CI 0.07–0.16, compare second and third bars in Fig. 5b) and FRIC versus nFRIC_bias_ (*P* < 0.001, median difference 0.12, 95% CI 0.08–0.15, compare second and fourth bars in Fig. 5b).

Similarly, we found significant differences in the number of FRIC members detected when lesioning the original empirical FRIC members compared with empirical non-FRIC members (*P* < 0.001, median difference 11.51, 95% CI 11.50–11.52, compare first and second bars in Fig. 5c), Emp versus nFRIC (*P* < 0.001, median difference 6.63, 95% CI 4.03–9.23, compare first and third bars in Fig. 5c) and Emp versus nFRIC_bias_ (*P* < 0.001, median difference 3.63, 95% CI 2.04–5.22, compare first and fourth bars in Fig. 5c). We also compared FRIC versus nFRIC (*P* < 0.001, median difference 4.88, 95% CI 2.27–7.49), compare second and third bars in Fig. 5c) and FRIC versus nFRIC_bias_ (*P* < 0.001, median difference 7.88, 95% CI 6.27–9.49, compare second and fourth bars in Fig. 5c). This provides mechanistic evidence for the role of FRIC in orchestrating functional organization.

### Alternative measurement of hierarchy

**Extended Data Fig. 8:**
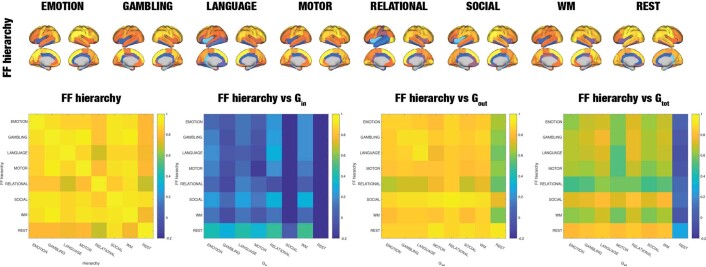
Alternative measure of hierarchy. Inspired by the neuroanatomical research by Markov and colleagues, we computed a similar measure of hierarchy as the fraction of feedforward and feedback (FF) organisation. We computed this for the seven tasks and for resting state (top row). As can be seen from the correlation matrices, this FF Hierarchy measure is similar to G_out_ for all seven tasks (mean correlation is 0.81 and the mean p-values are p < 0.001). On the other hand FF Hierarchy is only non-significantly correlated with G_in_, the integrative measure of incoming information flow (mean correlation is −0.02 and the mean p-value is p = 0.243).

In addition to the NDTE measures of connection strength, Extended Data Fig. 8 shows the result of measuring another hierarchical measurement inspired by the seminal generalized linear model (GLM) method for computing the fraction of feedforward and feedback (FF) hierarchical organization, which was originally developed in neuroanatomy^1,3,41^ (see Methods).

## Discussion

**Extended Data Fig. 9:**
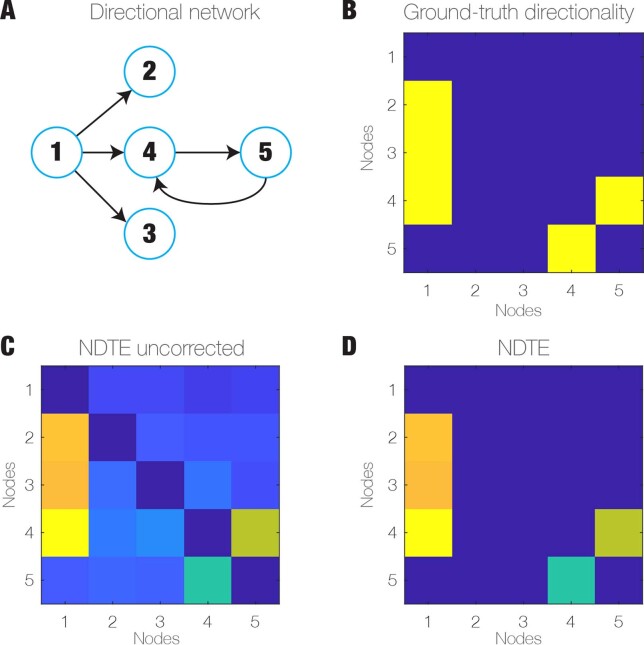
Validation of NDTE framework. Following the work of Seth, Chorley and Barrett^1^, we constructed a directional network with five nodes and simulated BOLD timeseries by using a Hopf model. **b**, This network is represented by the simple directional coupling 5×5 matrix. The generative Hopf model was run 10 times with 1200 timepoints sampled at TR = 0.72 s. We then validated the NDTE framework by applying this to the simulated data (using the same lag of 10 and 100 surrogates as in the analysis of the empirical data). **c**, The figure shows the recovered NDTE uncorrected matrix without using surrogates and aggregation of p-values. **d**, In contrast, the figure shows the results of using surrogates and aggregation of p-values and corrected for multiple comparisons. The NDTE framework is clearly able to recover the original couplings.

The acquisition of large multimodal neuroimaging datasets (Big Data) is now making it possible to address fundamental problems in systems neuroscience^18,42,43^. A key open problem is how best to characterize the functional hierarchical organization of whole-brain dynamics to understand the orchestration of brain processing. We quantified the ‘GW’^14,15^ as the core common FRIC regions invariant across seven tasks and rest in 1,003 participants^19^. To estimate the overall functional hierarchical organization of the human brain and the corresponding regions in the FRIC in resting state and across seven tasks, we developed an NDTE framework, validated through retesting (Extended Data Fig. 3) and external validation (see Extended Data Fig. 9 and Methods). This provided the possibility to precisely characterize functional hierarchical organization by estimating the flow of information between pairs of brain regions. Finally, the importance of the findings was confirmed by building and lesioning a whole-brain model, which not only gave a mechanistic understanding of the emergence of the FRIC but also showed that the regions in the FRIC are orchestrating brain function.

The GW was found to consist of a core subset of brain regions including the precuneus, posterior and isthmus cingulate, nucleus accumbens, putamen, hippocampus and amygdala. This core functional ‘club’ of integrative brain regions is consistent with the original proposal by Dehaene and Changeux^15^, which suggests that the global neuronal workspace must integrate past and present through focusing and evaluation. Indeed, Dehaene and Changeux proposed that associative perceptual, motor, attention, memory and value areas interconnect to form a higher-level unified space. For the integration of the past, the hippocampus has been shown to play a key role in many aspects of memory (see, for example, refs. ^44–46^). Similarly, the evaluation of value has been shown to involve the nucleus accumbens (see, for example, refs. ^47–49^), putamen (see, for example, refs. ^49,50^) and amygdala (see, for example, refs. ^49,51–54^). The integration of the past, present and future by processing and attending perceptual information has been strongly associated with the precuneus (see, for example, refs. ^55–57^) and the posterior and isthmus cingulate cortices (see, for example, refs. ^49,57–60^). Interestingly, the functions of the precuneus have also been shown to be compromised in coma and vegetative state^61^.

Baars’ cognitive theory of consciousness distinguishes a vast array of unconscious specialized processors running in parallel, and a single limited-capacity serial GW that allows them to exchange information^14^. The subsequent development by Dehaene and Changeux^62^ of this theory into the global neuronal workspace includes a further ‘ignition’ component capturing the strong temporary increase in synchronized firing leading to a coherent state of activity. The transition to this state of highly correlated activity is very fast and leads to amplification of local neural activation and the subsequent ignition of multiple distant areas. We have not studied ignition here since the fast timescale (typically about 100–200 ms) is difficult to capture with fMRI—although we have recently shown that such fast timescales can be reconstructed using appropriate whole-brain modelling^63^. Yet, the potential role of ignition in initiating and sustaining FRIC and the GW should clearly be further investigated in future studies using, for example, MEG.

The identification of the GW using whole-brain modelling of the established information flow (with NDTE) in the empirical data complements existing methods for establishing information^20–22^ and functional gradiental hierarchy^8,64^.

Our findings are consistent with the gradiental perspective on hierarchical processing^8,64^ but goes beyond these findings due to the ability of our NDTE framework to estimate bidirectional information flow in both resting and tasks. We note that, even though we have used the most comprehensive dataset currently available with rest and tasks covering a broad cognitive domain, in the future it would be of considerable interest to compare the results in an even larger set of tasks and participants.

A long history of neuroanatomical discoveries has demonstrated that the brain is clearly hierarchical in its structure, from single units to the larger circuits^1–7^. Research by Margulies and colleagues^8^ used neuroimaging to extend Mesulam’s seminal proposal that brain processing is shaped by a hierarchy of distinct unimodal areas to integrative transmodal areas^2^. Recently, this idea has been further extended by applying the principle of harmonic modes to functional connectivity HCP data^64^.

We extend these findings using the advanced NDTE framework to establish bidirectional information flow for identifying the functional hierarchical organization of the human brain. We have demonstrated that this can be used to characterize the FRIC corresponding to the core integrative transmodal brain regions allowing for the necessary whole-brain cohesion (as shown by the cartoon in a simplified 2D representation in Fig. 1c). As shown in Fig. 2, the outgoing information flow (drivers), *G*_out_, reflects mostly the unimodal sensory regions, whereas the incoming information flow (integrative receivers), *G*_in_, reflects the higher-order transmodal regions.

As shown by our results here, the NDTE framework can be used for any spatial scale and yields very consistent results from the three parcellations we have used here (DK80, Brainnetome246 and Glasser360). Still, it is important to note that there is no current consensus about what is the correct spatial parcellation scheme, as shown by a recent paper by Eickhoff and colleagues reviewing the literature on the topographic organization of the brain^65^. Nevertheless, the results are similar and consistent at the macroscale, reflecting the functional organization of the human brain at different spatial scales.

Furthermore, we also confirmed the results by applying the NDTE framework to MEG time series from the 62 cortical regions of the DK80 parcellation, and demonstrating similarly high correlations with myelination (Extended Data Fig. 2). This clearly demonstrates the robustness of the NDTE framework for both haemodynamic and direct electromagnetic measures of brain activity.

The NDTE framework includes suitable normalization, surrogates and *P* value aggregation across large number of participants (see Methods), which contribute to the robustness of the method. Indeed, the high correlation between myelination and the total incoming and outgoing information flow, *G*_tot_, obtained for both blood-oxygen-level-dependent (BOLD) and MEG signals, provides further confidence in the results.

To provide further evidence of the causal significance of the core FRIC regions, we built and lesioned a whole-brain model that can generate effective connectivity obtained using our NDTE framework (see Fig. 5 and Methods). We found that lesioning ten FRIC regions for the resting state very significantly impaired the flow of information and the ability to form new FRIC among the remaining brain regions. This causally establishes the full FRIC network as having a key integrative role in orchestrating functional hierarchical organization. Furthermore, we found that this FRIC network is fairly fault tolerant in that lesioning four FRIC members was not significantly different from lesioning four non-FRIC members. However, lesioning five FRIC compared with five non-FRIC members did lead to significantly different information flow. This suggests that there is a tipping point beyond which the breakdown of information flow in the FRIC leads to important problems. It is possible that the partial breakdown of FRIC members could be a considerable factor in the transitioning to neuropsychiatric disorders^66^. In particular, this leads to the speculation that the specific breakdown of the integration of the evaluative system related to self-processing (precuneus, nucleus accumbens, putamen and cingulate cortices) could lead to the anhedonia, the lack of pleasure, which is a major symptom of neuropsychiatric disease^67,68^.

Traditionally, whole-brain models have been relatively successful in linking SC with functional dynamics^66,69,70^. This has revealed important mechanistic principles of brain function^63,71–74^. In particular, the Hopf model has been shown to be useful for modelling both fMRI^73,75–77^ and MEG functional connectivity data^78^. In fact, we chose the Stuart–Landau Hopf model, since it has been shown that this is the most universal non-linear oscillator, being applied broadly across a large set of completely different physical systems^79^.

The findings presented here help shed light on a major unsolved problem in neuroscience. While the results presented here pertain to the GW of conscious processing, future work could use the NDTE framework to investigate other states such as sleep and anaesthesia, allowing for direct comparison with other theories of consciousness^80,81^. Equally, the NDTE framework could be used to investigate unbalanced brain states in neuropsychiatric disorders and be used to perturb and rebalance the model to identify novel optimal, causal paths to health^66,73,82^.

## Methods

### Neuroimaging data acquisition, preprocessing and time series extraction

#### Ethics

The Washington University–University of Minnesota (WU-Minn HCP) Consortium obtained full informed consent from all participants, and research procedures and ethical guidelines were followed in accordance with Washington University institutional review board approval (Mapping the Human Connectome: Structure, Function and Heritability; IRB # 201204036).

#### Participants

The dataset used for this investigation was selected from the March 2017 public data release from the Human Connectome Project (HCP), where we chose a sample of 1,003 participants, all of whom having resting-state data. For the seven tasks, HCP provides the following numbers of participants: working memory (WM), 999; SOCIAL, 996; MOTOR, 996; LANGUAGE, 997; GAMBLING, 1,000; EMOTION, 992; RELATIONAL, 989. We also validated the framework in the 45 participants with retest data. No statistical methods were used to pre-determine sample sizes, but our sample sizes are similar to those reported in previous publications using the full HCP dataset.

#### Neuroimaging acquisition for fMRI HCP

The 1,003 HCP participants were scanned on a 3-T connectome-Skyra scanner (Siemens). We used one resting-state fMRI acquisition of approximately 15 min acquired on the same day, with eyes open with relaxed fixation on a projected bright cross-hair on a dark background as well as data from the seven tasks. The HCP website (http://www.humanconnectome.org/) provides the full details of participants, the acquisition protocol and preprocessing of the data for both resting state and the seven tasks. Below we have briefly summarized these.

#### Neuroimaging acquisition for dMRI HCP

We obtained multi-shell diffusion-weighted imaging data from 985 subjects of the HCP 1200 data release. The standard acquisition protocol takes 59 min (six runs, each of approximately 9 min 50 s). We also obtained diffusion spectrum and T2-weighted imaging data from 32 participants from the HCP database who were scanned for a full 89 min. The acquisition parameters for both groups are described in detail on the HCP website^83^.

#### Neuroimaging acquisition for MEG HCP

We used the human non-invasive resting-state MEG data publicly available from the Human Connectome Project (HCP) consortium, acquired on a Magnes 3600 MEG (4D NeuroImaging) with 248 magnetometers. The resting-state data consist of 89 subjects (mean 28.7 years, range 22–35 years, 41 female/48 male, acquired in three subsequent sessions lasting 6 min each).

#### The HCP task battery of seven tasks

The HCP task battery consists of seven tasks: working memory, motor, gambling, language, social, emotional and relational, which are described in detail on the HCP website^40^. HCP states that the tasks were designed to cover a broad range of human cognitive abilities in seven major domains that sample the diversity of neural systems: (1) visual, motion, somatosensory and motor systems; (2) working memory, decision-making and cognitive control systems; 3) category-specific representations; (4) language processing; (5) relational processing; (6) social cognition; (7) emotion processing. In addition to resting-state scans, all 1,003 HCP participants performed all tasks in two separate sessions (first session: working memory, gambling and motor; second session: language, social cognition, relational processing and emotion processing). As a test–retest control condition, a small subsample of 45 HCP participants performed the paradigm twice.

### Neuroimaging SC and extraction of functional time series

#### Parcellations

All neuroimaging data were processed using two standard cortical parcellations with added subcortical regions. For a fine-scale parcellation, we used the Glasser parcellation with 360 cortical regions (180 regions in each hemisphere)^28^. We added the 18 subcortical regions, that is 9 regions per hemisphere: hippocampus, amygdala, subthalamic nucleus (STN), globus pallidus internal segment (GPi), globus pallidus external segment (GPe), putamen, caudate, nucleus accumbens and thalamus. This created a parcellation with 378 regions, that is, the Glasser378 parcellation, which is defined in the common HCP Connectivity Informatics Technology Initiative (CIFTI) grayordinates standard space with a total of 91,282 grayordinates (sampled at 2 mm^3^).

For a coarser-scale parcellation, we used the Mindboggle-modified Desikan–Killiany parcellation^30^ with a total of 62 cortical regions (31 regions per hemisphere)^31^. We added the same 18 subcortical regions mentioned above (9 regions per hemisphere) and ended up with 80 regions in the DK80 parcellation, also precisely defined in the common HCP CIFTI grayordinates standard space.

For a medium-scale parcellation, we used the Brainnetome parcellation with a total of 246 regions including 210 cortical regions (105 regions per hemisphere) and 36 subcortical regions (18 per hemisphere)^29^. Finally, to compare the incoming and outgoing information flow with existing networks, we used the very coarse-scale Yeo parcellation with seven regions^84^ (see Supplementary Fig. 1).

#### Generating SC matrices from dMRI

To be as precise as possible for the model fitting, we estimated the SC matrix from two HCP dMRI datasets. The first dataset, standard HCP dMRI (http://www.humanconnectome.org/), uses the highest quality multi-shell diffusion data acquired in sequence, taking 59 min from 985 HCP participants (HCP data acquired at Washington University in St. Louis; see Acknowledgements)^19,85^ (for HCP specifications, see their website). The second dataset, special HCP dMRI (http://www.humanconnectome.org/), uses even better protocols, taking 89 min for each of 32 HCP participants at the Massachusetts General Hospital centre. Both dMRI datasets were preprocessed and made available as part of the freely available Lead-DBS software package (http://www.lead-dbs.org/).

The precise preprocessing is described in detail in Horn and colleagues^86^, but briefly, the data were processed using a generalized *q*-sampling imaging algorithm implemented in DSI Studio (http://dsi-studio.labsolver.org). Segmentation of the T2-weighted anatomical images produced a white-matter mask and co-registering of the images to the b0 image of the diffusion data using SPM12. In each HCP participant, 200,000 fibres were sampled within the white-matter mask. Fibres were transformed into Montreal Neurological Institute (MNI) space using Lead-DBS^87^. The methods used the algorithms for false-positive fibres shown to be optimal in recent open challenges^88,89^. The risk of false-positive tractography was reduced in several ways. Most importantly, this used the tracking method achieving the highest (92%) valid connection score among 96 methods submitted from 20 different research groups in a recent open competition^88^. We subsequently used the standardized methods in Lead-DBS to produce the structural connectomes for the DK80 parcellation used in the whole-brain Hopf model (see section ”Whole-brain model of NDTE flow”).

#### Preprocessing and extraction of functional time series in fMRI resting state and task data

The preprocessing of the HCP resting state and task datasets is described in detail on the HCP website. Briefly, the data are preprocessed using the HCP pipeline, which is based on standardized methods using FSL (FMRIB Software Library), FreeSurfer and the Connectome Workbench software^90,91^. This standard preprocessing included correction for spatial and gradient distortions and head motion, intensity normalization and bias field removal, registration to the T1-weighted structural image, transformation to the 2-mm MNI space, and using the FIX artefact removal procedure^91,92^. The head motion parameters were regressed out and structured artefacts were removed by independent component analysis followed by FMRIB’s ICA-based X-noiseifier (ICA+FIX) processing^93,94^. Preprocessed time series of all grayordinates are in HCP CIFTI grayordinates standard space and available in the surface-based CIFTI file for each participants for resting state and each of the seven tasks.

We used a custom-made MATLAB script using the ‘ft_read_cifti’ function (Fieldtrip toolbox^95^) to extract the average time series of all the grayordinates in each region of the Glasser378, Brainnetome246 and DK80 parcellations, which are defined in the HCP CIFTI grayordinates standard space. The BOLD time series were filtered using a second-order Butterworth filter in the range of 0.008–0.08 Hz.

#### Preprocessing and extraction of MEG data time series

For each participant, the MEG data were acquired in a single continuous run comprising resting state. As a starting point, we used the preprocessed MEG data from the HCP database. At this level of preprocessing, removal of artefactual independent components, bad samples and channels has already been performed. Following the preprocessing pipeline detailed in ref. ^96^, MEG data were then downsampled to 250 Hz using an anti-aliasing filter, filtered to remove frequencies below 1 Hz, co-registered with the head models provided by the HCP and source-reconstructed using linearly constrained minimum-variance beamforming^97^ to the 62 cortical regions of the DK80 parcellation. For each region, a single time series was computed as the first principal component of the voxels within that parcel. Each region’s time series was then standardized to have mean of zero and standard deviation of one, so that the amount of variance was always the same regardless of the depth of the region. To project the results to brain space, we used a weighted mask, where each region had its maximum value at the centre of gravity.

We did not apply correction for spatial leakage (volume conduction), because the amount of signal removal would have been too large given the number of regions in the parcellation, thus making the rest of the analysis impossible. Although the similarity with the fMRI results suggests that the impact of signal leakage is not too disruptive for the conclusions of this study, this remains a limitation of this work, and future efforts will be dedicated to rigorous assessment of the impact of different approaches to leakage correction on the NDTE metric, as well as to the consideration of ‘softer’ versions of such leakage correction approaches that can be used in this case.

### Neuroimaging analysis tools and methods

#### Normalized directed transfer entropy

To establish and investigate the functional hierarchical organization of whole-brain activity, we first need to characterize how different brain regions communicate with each other, that is, to compute the directed flow between regions. We characterize the functional interaction between two brain regions, in a given parcellation, by an information-theoretical statistical criterion that allows us to infer the underlying bidirectional reciprocal communication. The NDTE framework was inspired by the work of Brovelli and colleagues, who used and validated a similar transfer entropy framework for neuroimaging data^25^. This framework uses a Gaussian approximation, that is, only second-order statistics of the involved entropies, which means, as shown below, that instead of estimating the probabilities, the method estimates the covariance, which massively facilitates computation. Finally, as also outlined below, we add four key elements to this powerful framework: normalization, multiple timepoints in the past, circular surrogates and aggregation of *P* values to improve the reliability and robustness of the NDTE framework.

Let us assume that we want to describe the statistical causal interaction exerted from a source brain area *X* to another target brain area *Y*. We aim to measure the extra knowledge that the dynamical functional activity of the past of *X* contributes to the prediction of the future of *Y*, by the following mutual information:

1 $$I\left( {Y_{i + 1};X^i|Y^i} \right) = H\left( {Y_{i + 1}|Y^i} \right) - H\left( {Y_{i + 1}|X^i,Y^i} \right)$$

where *Y*_*i*+1_ is the activity level of brain area *Y* at time point *i* + 1, and *X*^*i*^ indicates the whole activity level of the past of *X* (filtered BOLD signal sampled in rpetition time (TR)) in a time window of length *T* up to and including the time point *i* (that is, *X*^*i*^ *=* [*X*_*i*_ *X*_*i*-1_ *… X*_*i*-(*T*-1)_]). Following Kantz and Schreiber^98^, we estimated the autocorrelations of the empirical BOLD signals for all regions and all participants, finding that on average this decay to the first minimum corresponded to a value of *T =* 10. Note that this causality measure is not symmetric, which allows bidirectional analysis. The conditional entropies are defined as follows:

2 $$\begin{array}{l}H\left( {Y_{i + 1}{\mathrm{|}}Y^i} \right) = H\left( {Y_{i + 1},Y^i} \right) - H\left( {Y^i} \right)\\ = - \mathop {\sum}\nolimits_{y_{i + 1},y^i} {p\left( {y_{i + 1},y^i} \right){\mathrm{log}}\left( {p\left( {y_{i + 1}|y^i} \right)} \right)} \end{array}$$

3 $$\begin{array}{l}H\left( {Y_{i + 1}{\mathrm{|}}X^i,Y^i} \right) = H\left( {Y_{i + 1},Y^i,X^i} \right) - H\left( {X^i,Y^i} \right)\\ = - \mathop {\sum}\nolimits_{y_{i + 1},y^i,x^i} {p\left( {y_{i + 1},y^i,x^i} \right){\mathrm{log}}\left( {p\left( {y_{i + 1}|x^i,y^i} \right)} \right)} \end{array}$$

The mutual information *I*(*Y*_*i*+1_*; X*^*i*^*|Y*^*i*^) expresses the degree of statistical dependence between the past of *X* and the future of *Y*. In other words, if that mutual information is equal to zero, then the probability $p\left( {Y_{i + 1},X^i{\mathrm{|}}Y^i} \right) = p\left( {Y_{i + 1}|Y^i} \right).p\left( {X^i|Y^i} \right)$, and thus we can say that there is no causal interaction from *X* to *Y*.

Consequently *I*(*Y*_*i*+1_*; X*^*i*^*|Y*^*i*^) expresses a strong form of Granger causality^99^, by comparing the uncertainty in *Y*_*i*+1_ when using knowledge of only its own past *Y*^*i*^ or the past of both brain regions, that is, *X*^*i*^, *Y*^*i*^. This information-theoretical concept of causality was introduced in neuroscience by Schreiber^100^ and is usually called the transfer entropy^25,101–103^. To facilitate computation, Brovelli et al.^25^ proposed a weaker form of causality allowing the calculation of the involved entropies by just considering a Gaussian approximation, that is, by considering only second-order statistics. Indeed, under this approximation, the entropies can be computed as follows:

4 $$H\left( {Y^i} \right) = \frac{T}{2}\log \left( {2\pi e} \right) + \frac{1}{2}{\mathrm{log}}\left( {\det \left( {{{\Sigma }}(Y^i)} \right)} \right)$$

5 $$H\left( {Y_{i + 1},Y^i} \right) = \frac{{T + 1}}{2}\log \left( {2\pi e} \right) + \frac{1}{2}{\mathrm{log}}\left( {\det \left( {{{\Sigma }}(Y_{i + 1},Y^i)} \right)} \right)$$

6 $$H\left( {X^i,Y^i} \right) = T\log \left( {2\pi e} \right) + \frac{1}{2}{\mathrm{log}}\left( {\det \left( {{{\Sigma }}(X^i,Y^i)} \right)} \right)$$

7 $$H\left( {Y_{i + 1},Y^i,X^i} \right) = \frac{{2T + 1}}{2}\log \left( {2\pi e} \right) + \frac{1}{2}{\mathrm{log}}\left( {\det \left( {{{\Sigma }}(Y_{i + 1},Y^i,X^i)} \right)} \right)$$

In other words, causality is based only on the corresponding covariance matrices.

To be able to sum and compare the directed mutual information flow between different pairs of brain regions, this has to be appropriately normalized. In fact, if the mutual information directed flow is correctly normalized, then the different values could be combined, for example, to determine the total directed flow exerted by the whole brain on a single region or, vice versa, the directed flow exerted by a single brain region on the whole brain. More specifically, we define this information-theoretical measure as the NDTE flow *F*_*XY*_ from time series *X* to *Y*:

8 $$F_{XY} = I\left( {Y_{i + 1};X^i|Y^i} \right)/I\left( {Y_{i + 1};X^i,Y^i} \right)$$

where $I\left( {Y_{i + 1};X^i,Y^i} \right)$ is the mutual information that the past of both signals together, *X*^*i*^, *Y*^*i*^, has about the future of the target brain region *Y*_*i*+1_. Given that

9 $$I\left( {Y_{i + 1};X^i,Y^i} \right) = I\left( {Y_{i + 1};Y^i} \right) + I\left( {Y_{i + 1};X^i|Y^i} \right)$$

this normalization compares the original mutual information directed flow, that is, the predictability of *Y*_*i*+1_ by the past of *X*^*i*^|*Y*^*i*^, with the internal predictability of *Y*_*i*+1_, that is *I*(*Y*_*i*+1_;*Y*^*i*^).

We define the matrix of NDTE flow in the brain for a given parcellation with *N* regions, ${\cal{C}}$, with elements ${\cal{C}}_{ij} = F_{X\left( i \right)X(j)}$, where *X*(*k*) corresponds to the filtered BOLD time series from region *k*.

**Extended Data Fig. 10:**
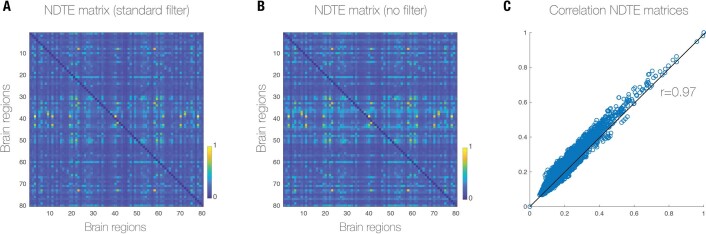
The estimation of the NDTE matrix does not depend on the filtering. **a**, We computed the NDTE matrix in the DK80 parcellation from 100 unrelated HCP participants using a standard filter of 0.01-0.2 Hz on the underlying BOLD data. **b**, Similarly, we computed the NDTE matrix for the same participants with no filter. **c**, The correlation between the two NDTE matrices for the filtered and non-filtered version is almost identical (r(3160)=0.972, P < 0.001, 95% CI = 0.971–0.973).

We note that it has been shown that transfer entropy can be modulated and influenced by the frequencies and bandwidth of the filter of high-temporal-resolution signals coming from MEG/electroencephalography^20,104^, but these potential obstacles related to the high temporal resolution are insignificant for the slow, narrowly filtered (0.008–0.08 Hz) BOLD signal. We show that the NDTE DK80 matrices for the filtered and non-filtered version of resting state for 100 unrelated HCP participants show a correlation of 0.97 (Extended Data Fig. 10).

Furthermore, to perform a statistical significance analysis of the NDTE flow, ${\cal{C}}$, we use the surrogate framework, inspired by the work of Theiler and colleagues^105^. This traditional surrogate methodology uses a phase randomization of the Fourier transform of the original data to preserve the linear correlations. Nevertheless, as discussed and analysed rigorously in Diks and Fang^106^; these methods are not suitable for detecting significance when using entropy measures, as discussed extensively^107,108^. In view of these problems, Quiroga and colleagues proposed the circular time-shifted surrogates method, which is a method using surrogates that can be used for causality measurements^23^.

There have been many suggestions of how best to construct surrogate data; see, for example, ref. ^109^. A first approach is to construct time series with the same power spectrum (that is, an autocorrelation function) of the original ones, but with chance cross correlation. A more refined approach is to construct surrogates that also maintain the amplitude distribution (AAFT)^110^. However, this is based on an iterative procedure that does not always converge. In contrast, in ref. ^27^ a very simple solution was proposed: to shift one dataset with respect to the other. This way, the properties (amplitude and autocorrelation spectrum, etc.) of each signal are maintained because the signals are exactly the same, but the cross-correlation is reduced to chance levels, which is exactly what is required, as the hypothesis tested is whether a synchronization or directionality value is larger than chance. Furthermore, the use of time-shifted surrogates has already been shown to provide optimal results with several linear and non-linear synchronization measures^27,109^.

Hence, we use the circular shift methodology for analysing the *P* values of the hypothesis testing, aiming to detect significant values in ${\cal{C}}$ for each pair for each single participant. For each statistical test (that is, each pair of regions and each subject), we generate 100 independent circular time-shifted surrogates by separately resampling both the driving signal *X* and the target response signal *Y*. Specifically, two independent integers *c* and *d* are randomly generated within the interval [0.05*n* 0.95*n*] (where *n* is the number of time points in the time series signal). Then, the circular time shift is performed by moving the first *c* values of *X* = [*X*_1_,…*X*_*n*_] to the end of the time series, which creates the surrogate sample $X = [X_{c + 1}, \ldots ,X_n,X_1, \ldots ,X_c]$, and similarly for *Y* the first *d* values of $Y = [Y_1, \ldots ,Y_n]$ are moved to the end of the time series to create the surrogate sample $Y = [Y_{d + 1}, \ldots ,Y_n,Y_1, \ldots ,Y_d]$.

This type of surrogate does not assume Gaussianity and preserves the whole statistical structure of the original time series. We use a nonparametric kernel distribution representation of the probability density function of the surrogate values of ${\cal{C}}$, and compare the fraction of area of that distribution above the value of the NDTE flow of the original data, ${\cal{C}}_{original}$, with the total area, and compute the corresponding *P* value. Supplementary Fig. 2 shows all the *P* values for all the pairs of time series across all the participants in each of the eight conditions coming from the surrogates.

After computing the individual *P* values for each brain region pair and each single participant, we aggregate the *P* values for each single pair of brain areas across the whole group of participants. The combination of different *P* values across subjects is a classical problem in statistics that was originally addressed by Ronald Fisher in what is nowadays known as Fisher’s method^111^. Here, we used a more sensitive methodology, namely the Stouffer’s method^24^, which sums the inverse normal transformed *P* values. Indeed, the Stouffer’s statistic is given by

10 $$S = \mathop {\sum}\nolimits_i {{{\Phi }}^{ - 1}(p_i)}$$

where Φ is the standard normal cumulative distribution function, and the _*pi*_ are the *P* values of each participant *i* (computed for a given pair). Under the null hypothesis, the Stouffer’s statistic is normally distributed *N*(0, *m*), where *m* is the total number of participants. After the aggregation of the pairs of *P* values across participants, we correct for multiple comparisons by using the traditional false discovery rate method of Benjamini and Hochberg^112^. We call the resulting NTDE matrix ${\cal{C}}_{\mathrm{All}}$.

The result of the significance test across participants determines a binary matrix *T* (with the dimension being the number of brain regions in a given parcellation) that indicates with ones or zeros whether the corresponding pair is significant or not (rows indicate target regions, and columns driving regions).

#### Validation of NDTE framework

First, to show that our NDTE framework captures the main causal connectivity of a given network, we use the framework and network proposed by Seth, Chorley and Barrett^113^. Our generative Hopf model (described in detail below) simulated BOLD signals in the proposed coupled network, which is shown graphically in Extended Data Fig. 9a and in binary matrix form in Extended Data Fig. 9b. The model used the same parameters for all the nodes as specified below in the description of the Hopf model, except using a bifurcation parameter of *a* = −0.2 and a global coupling strength parameter of *G* = 0.13. We generated time series of 1,200 points sampled with the same TR as the empirical data (0.72 s) and aggregated the run from ten repetitions. The NDTE framework was applied to the simulated data (using the same lag of 10 and 100 surrogates as in the analysis of the empirical data).

This validated the framework by recovering the causal directionality of the underlying network (compare Extended Data Fig. 9b with Extended Data Fig. 9d). We note that, for correct estimation, it is clearly important to use the three new key elements that we have added to the transfer entropy framework: normalization, circular surrogates and aggregation of *P* values (compare the results in Extended Data Fig. 9d with the results without these extra ingredients in Extended Data Fig. 9c).

In terms of further validation, it is important to note that there have already been important validations of the transfer entropy framework. Most importantly, it has already been shown that using surrogates is essential to avoid problems with determining directionality^26^. It is well known that, rather than measuring actual causality, directionality measures can reflect intrinsic properties of each of the signals. In particular, research has shown that directionality can even be reversed, which depends mainly on the noise level and the main frequencies of each of the signals. However, a solution to this problem was proposed by Quiroga^27^, who showed that such bias can easily be detected by using surrogates and that causality can be established using the time-shifted surrogates that we use. Another important validation is that the use of the time-shifted surrogates has been shown to give optimal results with several linear and non-linear synchronization measures^27,109^.

#### Functional hierarchical organization

Using the above methods describing NDTE flow, we can establish and study the functional organization of data where the different levels of directed flow to and from a given brain region *i* are given by *G*_in_(*i*), *G*_out_(*i*), and *G*_tot_(*i*).

Our analysis of functional relevance and hierarchy is based on the resulting averaged NDTE flow,${\cal{C}}_{\mathrm{All}}$, across all participants. We define for each brain region *i* the incoming level of directed flow, that is, the degree of being a receiver, by $G_{\mathrm{in}}(i) = \mathop {\sum}\nolimits_j {{\cal{C}}_{{\mathrm{All}}_{ij}}}$. Similarly, for each brain region *i* the outgoing level of NDTE flow, that is, the degree of being a driving region, is defined by $G_{\mathrm{out}}(i) = \mathop {\sum}\nolimits_j {{\cal{C}}_{{\mathrm{All}}_{ji}}}$. The total level of functional interaction for each brain region *i* is thus given by $G_{\mathrm{tot}}\left( i \right) = G_{\mathrm{in}}\left( i \right) + G_{\mathrm{out}}(i)$.

#### Functional rich club

We define the FRIC in a matrix ${\cal{C}}_{\mathrm{All}}$ with *N* regions by running a simple algorithm that searches for the largest subset of regions $k = [i_1,..,i_l]$, where *G*_FRIC_(*k*) is significantly larger than all other sets with the same number of regions:

11 $$G_{\mathrm{FRIC}}\left( k \right) = \mathop {\sum}\nolimits_k {{\cal{C}}_{{\mathrm{All}}_k}} + \mathop {\sum}\nolimits_k {G_{\mathrm{in}}\left( k \right)} - \mathop {\sum}\nolimits_k {G_{\mathrm{out}}\left( k \right)}$$

Using a more detailed notation, the equation can be further expressed as $G_{\mathrm{FRIC}}\left( k \right) = \mathop {\sum}\nolimits_{i = 1}^l {\mathop {\sum}\nolimits_{j = 1}^l {{\cal{C}}_{{\mathrm{All}}_{ij}}} } + \mathop {\sum}\nolimits_{i = 1}^l {G_{\mathrm{in}}\left( i \right)} - \mathop {\sum}\nolimits_{j = 1}^l {G_{\mathrm{out}}\left( j \right)}$, where *l* is the number of regions that defines the subset of regions *k* = [*i*_1_,…*i*_*l*_].

The first and third term can be transformed to

$$\mathop {\sum }\limits_{i = 1}^l \mathop {\sum }\limits_{j = 1}^l {\cal{C}}_{{\mathrm{All}}_{ij}} - \mathop {\sum }\limits_{j = 1}^l G_{\mathrm{out}}(j) = \mathop {\sum }\limits_{j = 1}^l \left[ {\mathop {\sum }\limits_{i = 1}^l {\cal{C}}_{{\mathrm{All}}_{ij}} - G_{\mathrm{out}}(j)} \right] = - \mathop {\sum }\limits_{j = 1}^l \mathop {\sum }\limits_{i = l + 1}^N {\cal{C}}_{{\mathrm{All}}_{ij}}$$

where *N* is the total number of regions.

Similarly, the second term, that is the sum of *G*_in_, can be expressed as

$$\mathop {\sum }\limits_{i = 1}^l G_{\mathrm{in}}(i) = \mathop {\sum }\limits_{i = 1}^l \mathop {\sum }\limits_{j = l}^N {\cal{C}}_{{\mathrm{All}}_{ij}} = \mathop {\sum }\limits_{j = 1}^l \mathop {\sum }\limits_{i = 1}^l {\cal{C}}_{{\mathrm{All}}_{ij}} + \mathop {\sum }\limits_{i = 1}^l \mathop {\sum }\limits_{j = l + 1}^N {\cal{C}}_{{\mathrm{All}}_{ij}}$$

Thus, taken together, Eq. 11 can also be written as

$$G_{\mathrm{FRIC}}\left( k \right) = \mathop {\sum }\limits_{j = 1}^l \left[ {\mathop {\sum }\limits_{i = 1}^l {\cal{C}}_{{\mathrm{All}}_{ij}} - \mathop {\sum }\limits_{i = l + 1}^N {\cal{C}}_{{\mathrm{All}}_{ij}}} \right] + \mathop {\sum }\limits_{i = 1}^l \mathop {\sum }\limits_{j = l + 1}^N {\cal{C}}_{{\mathrm{All}}_{ij}}$$

where the first term describes the difference between the information flow of each member of the subset to the other members in the subset compared with the information flow outside the subset. The second term describes the information flow that each member of the subset receives from outside.

As can be appreciated from the combinatorics, for a matrix ${\cal{C}}_{\mathrm{All}}$ with more than just a few regions, it is computationally very demanding to exhaustively compute the optimal solution. However, it is also clear from the definition that the FRIC for a given set of regions is likely to be found from the regions with the highest level of incoming directed flow (*G*_in_). So we created the following algorithm: Sort the regions according to *G*_in_, and then iteratively compute *G*_FRIC_ for progressively more *k* regions. Statistical significance was computed by replacing a random region of the *k* regions with any of the remaining regions using a Monte Carlo framework.

In more detail, the FRIC algorithm iteratively expands the FRIC by adding a new node to the existing club until the FRIC value of the FRIC club is no longer statistically significant by comparing whether a FRIC of length *k* has a significantly larger FRIC value than all other possible surrogate clubs of this length. The FRIC value is computed as the sum of the connections between all club members plus the sum of all the incoming connections for the club members minus the sum of all outgoing connections for the club members, as shown in Eq. 11. The FRIC value is also computed for all the permutations of all other clubs that can be made from the same number of existing nodes (with length *k*). As mentioned, given the challenges of the combinatorics, we used a Monte Carlo framework to generate the most similar and challenging permutations of the FRIC, that is, when comparing a potential new FRIC club of size *k*, the surrogate clubs (with *k* members) have the same *k* − *1* members with an added random new member. We run this procedure iteratively, starting from the top *G*_in_ regions, and continue while the *P* value of the comparison between the FRIC club and surrogates is smaller than 0.05. We seed the procedure with the top rich club region having the largest incoming NDTE flow.

As can be appreciated from the above description, the computational complexity of the FRIC algorithm is *NP* hard. Nevertheless, we wanted to estimate the GW in the finer parcellation of Brainnetome246. We therefore developed an approximation for estimating the GW, which computes the intersection of the top *G*_in_ regions across rest and seven tasks. This is only an approximation, since it does not conform fully to our definition of the GW, similar to a ‘club’ of functional hubs being characterized by a tendency to be more densely functionally connected among themselves than to other brain regions from which they receive integrative information. Nevertheless, this rough approximation shows an overlap with our FRIC algorithm, described in detail above.

Importantly, it should be noted that our definition of FRIC is different from the graph-theoretical concept of rich club, so we compared FRIC with the standard anatomical rich club for the SC^5,7^. Instead of applying the graph-theoretical rich club concept directly to the directed NDTE matrix, we designed the FRIC algorithm. Indeed, in graph theory, assortativity is the tendency of nodes of similar degree to connect to each other, while the concept of rich club measures the density of connectivity between high-degree nodes in an anatomical brain network^114^. The FRIC framework is an even more specific concept. designed to find our proposed quantification of the GW, namely a core set of brain regions, being similar to a ‘club’ of functional hubs characterized by a tendency to be more densely functionally connected among themselves than to other brain regions from where they receive integrative information and only give out sparsely (see Eq. 11). Still, future research could focus on improving this algorithm even further and investigate the temporal stability of FRIC and, for example, whether multiple clubs could co-exist over time.

#### Defining feedforward and feedback (FF) organizational hierarchy using GLM

In the literature, hierarchy has traditionally been proposed using neuroanatomy and specifically the anatomical hierarchical ordering based on the different structure of feedforward and feedback connections across the brain^1,115^. On this basis, Markov and colleagues defined a framework for assigning hierarchical values to each region in such a way that the difference of the hierarchical values in two brain regions predicts the fraction of feedforward connections coupling those two brain regions^3,41^. In fact, they proposed to use the supragranular layer neurons (SLN) index, defined as the fraction of projections originated in the supragranular layer of the source area to the target area divided by the total number of projections between the SLN of projections. This idea was based on the observations of Felleman and Van Essen^1^ and Barone and colleagues^115^ that, in the visual system, feedforward projections directed from early visual areas to higher-order areas tend to originate in the supragranular layers of the cortex and terminate in layer 4, whereas projections from higher-order areas to early visual areas originate in the infragranular layers and terminate outside of layer 4.

Our framework for computing the NDTE flow (see above) allows us to extend these seminal ideas to the functional level. Instead of using the anatomically based SLN index, we can use the fraction of functional feedforward connections with respect to the total number of connections, feedforward and feedback (FF), between two brain regions. Consequently, in this FF framework, we assign a functional hierarchical value *H* to each brain region such that the difference of the corresponding values between two brain regions predicts the functional fraction of direct connections from one source region *j* to another target region *i*. We use a GLM to establish this prediction, as follows:

12 $$\frac{{{\cal{C}}_{{\mathrm{All}}_{ij}}}}{{{\cal{C}}_{{\mathrm{All}}_{ij}} + {\cal{C}}_{{\mathrm{All}}_{ji}}}} = g^{ - 1}(H_i - H_j)$$

where the left-hand side is the fraction of feedforward connections with respect to the total, *g*^−1^ is a logistic regression function (which corresponds to fitting a GLM with a binomial family^116^) and *H*_*i*_ and *H*_*j*_ are the functional hierarchical values inferred for brain regions *i* and *j*. To establish a reference point, we assigned a hierarchy value of zero to the last parcel.

As shown in Extended Data Fig. 8, the FF hierarchy measure is similar to *G*_out_ in that it is not discriminatory between the seven tasks. On the other hand, it is only weakly correlated with *G*_in_, the integrative measure of incoming information flow needed for establishing FRIC. This is to be expected given that the FF hierarchy is an ordered measure of the fraction of feedforward and feedback connections, rather than a characterization of a given brain region in terms of incoming and outgoing information flow to/from the other regions in the brain, which is what is needed to establish a meaningful functional hierarchical organization. The results demonstrate that the FF hierarchy measure is not as useful for establishing the full hierarchical organization compared with the proposed NDTE framework.

#### Anatomical rich club

The members of the anatomical rich club were determined using the standard method with graph-theoretical tools^117^. A set of brain regions in the network shows a rich club structure if the level of interconnectivity exceeds the level of connectivity that can be expected on the basis of chance alone. The weighted rich club coefficient *Φ*(*k*) is computed as the ratio between (1) the weights of connections present within the subnetwork *S* of regions with degree > *k* and (2) the total sum of weights present within a subset of the same size of the top ranking connections in the network. The normalized rich club coefficient *Φ*_norm_(*k*) is computed by dividing *Φ*(*k*) by *Φ*_random_(*k*), with *Φ*_random_(*k*) computed as the average rich club coefficient for each *k* of a set of 1,000 randomized graphs (acquired by randomizing the adjacency matrix *A* while preserving the degree sequence)^118^. For a given region, membership to the rich club is determined if the normalized rich club coefficient *Φ*_norm_(*k*) is larger than one, for a range of increasing degree level *k*.

#### Whole-brain model of NDTE flow

The main aim of whole-brain modelling is to infer the dynamical mechanisms generating the observed empirical spatiotemporal dynamics. Here, we would like to estimate the generators underlying the empirical spatiotemporal dynamics in terms of the statistical relationships between the different brain regions, that is, the NDTE flow. Specifically, the whole-brain model will link the structural anatomy (given by the dMRI data) with the functional dynamics (given by the fMRI data) by adapting the free parameters, that is, the generators, to provide the optimal fit between the simulated and empirical NDTE flow. The generators are internal parameters describing the local dynamics of a brain region, such as noise and latency, as well as the strength of the synaptic conductivity of connections between different brain areas linked by the anatomical fibres. We call the matrix of the generative conductivities of the existing anatomical fibres the GABIC.

The GABIC matrix is estimated from the NDTE flow, which is bidirectional, and is therefore asymmetric. This is unlike the anatomical matrix extracted by dMRI tractography, which is unidirectional. Importantly, GABIC is defined as the generators accounting for the causal influence of one neural system over another.

While there is a large literature on dynamic causal modelling of effective connectivity within and among local neuronal (mass) models, the non-linear and emergent dynamical properties of these systems have yet to be explored thoroughly. Previous models used the unidirectional SC to reproduce functional connectivity (FC)^66,119^ and a common global conductivity value, meaning that only the scaling factor is optimized. In other words, the scaling factor is one global coupling parameter expressing the conductivity of all fibres equally. In contrast, another possibility is to tune network connections individually (but not independently), which requires a dedicated estimation procedure^120^. This corresponds to the concept of effective connectivity (EC)^121^, which describes how network nodes excite or inhibit each other for a given model of local dynamics. EC thus describes causal interactions whose effects are modulated by the local dynamic regime of the node, which may shape FC in a complex fashion^122^: two areas may be significantly correlated (in FC) although disconnected (zero EC) when strong indirect pathways connect them (that is, large network effect), as was shown by Robinson^123^. Biologically, EC measures the strengths of connections, which depend not only on anatomical properties embodied in SC values (connection densities) but also on heterogeneities in synaptic receptors or neurotransmitters. Gilson and colleagues provided a solution for estimating EC from fMRI FC with information about the directed connectivity at the whole-brain level (divided in a parcellation of 70 brain regions with a couple of thousand connections to tune).

Until now, there has not been a generative model that can explain the bidirectional flow of information across the whole brain, which is non-trivial given that the SC and NDTE matrices are not strongly correlated. Here, we extend existing models by using a more powerful bidirectional measure, namely the NDTE flow, that captures the underlying spatiotemporal dynamical mechanisms, and thus not correlations or time-shifted correlations. For that, we use a recent successful model, namely the Hopf whole-brain model^75^, in combination with a powerful non-gradient-based global optimization algorithm, namely particle swarm optimization.

Briefly in the following, we describe how whole-brain models aim to obtain a balance between complexity and realism to describe the most important features of the brain in vivo^124^. This balance is extremely difficult to achieve because of the astronomical number of neurons and the underspecified connectivity at the neural level. The emerging collective macroscopic dynamics of brain models use mesoscopic top-down approximations of brain complexity with dynamical networks of local brain area attractor networks^69,125^. Essentially, these models link anatomical structure (given by the dMRI tractography) and functional dynamics (typically measured with fMRI) to reproduce the whole-brain empirical data^71,126–128^.

Biologically informed models have been useful for modelling neuroimaging data, for example, MEG data^129^ and working points near the Hopf bifurcation. In fact, the emergence of functional brain networks from the combination of local dynamics and structural topology has also been captured by other models, including random walkers^130^, wave equations on a network^131^ and epidemic spreading models just beyond the critical epidemic threshold for analysing the information flow^132^.

Here, we use the Hopf whole-brain model consisting of coupled dynamical units (regions of interest or nodes) representing the *N* cortical and subcortical brain areas in a given parcellation^75^. The local dynamics of each brain region is described by the normal form of a supercritical Hopf bifurcation, also known as the Landau–Stuart oscillator, which is the canonical model for studying the transition from noisy to oscillatory dynamics^133^. Coupled together with the brain network architecture, the complex interactions between Hopf oscillators have been shown to reproduce important features of brain dynamics observed in electrophysiology^134,135^, MEG^136^ and fMRI^137,138^.

The dynamics of an uncoupled brain region *n* is given by the following set of coupled dynamical equations, which describe the normal form of a supercritical Hopf bifurcation in Cartesian coordinates:

13 $$\frac{{{\mathrm{d}x}_{{n}}}}{{{\mathrm{d}t}}} = \left[ {{{a}}_{{n}} - {{x}}_{{n}}^2 - {{y}}_{{n}}^2} \right]{{x}}_{{n}} - \omega _{{n}}{{y}}_{{n}} + \beta \eta _{{n}}({{t}})$$

14 $$\frac{{{\mathrm{d}y}_{{n}}}}{{{\mathrm{d}t}}} = \left[ {{{a}}_{{n}} - {{x}}_{{n}}^2 - {{y}}_{{n}}^2} \right]{{y}}_{{n}} + \omega _{{n}}{{x}}_{{n}} + \beta \eta _{{n}}({{t}})$$

where *η*_*n*_(*t*) is additive Gaussian noise with standard deviation *β*. This normal form has a supercritical bifurcation *a*_*n*_ = 0, so that, if *a*_*n*_ > 0, the system engages in a stable limit cycle with frequency *f*_*n*_ = *ω*_*n*_/2*π*. On the other hand, when *a*_*n*_ < 0, the local dynamics are in a stable fixed point representing a low-activity noisy state. Within this model, the intrinsic frequency *ω*_*n*_ of each region is in the 0.01–0.2 Hz band (*n* = 1, …, *N*), where *N* is the total number of regions. It has been shown that the optimized *a*_*n*_ form a distribution of negative and positive values, consistent with the heterogeneous mixture of regions at bifurcation, steady state or limit cycle, arising from the optimization of the power spectrum of the regions^75,76^.

We estimated the intrinsic frequencies from the empirical data, as given by the averaged peak frequency of the narrowband BOLD signals of each brain region. The variable *x*_*n*_ emulates the BOLD signal of each region *n*. To model the whole-brain dynamics, we added an additive coupling term representing the input received in region *n* from every other region *p*, which is weighted by the corresponding SC. The whole-brain dynamics was defined by the following set of coupled equations:

15 $$\begin{array}{ll}\frac{{{\mathrm{d}x}_{{n}}}}{{{\mathrm{d}t}}} = & \left[ {{{a}}_{{n}} - {{x}}_{{n}}^2 - {{y}}_{{n}}^2} \right]{{x}}_{{n}} - {\omega}_{{n}}{{y}}_{{n}} \\ &+ {{G}}\mathop {\sum}\nolimits_{{{p}} = 1}^{{N}} {{{C}}_{{{np}}}\left( {{{x}}_{{p}}({{t}} - {\tau}) - {{x}}_{{n}}} \right)} + \beta _n{\eta}_{{n}}\left( {{t}} \right)\end{array}$$

16 $$\begin{array}{ll}\frac{{{\mathrm{d}y}_{{n}}}}{{{\mathrm{d}t}}} = & \left[ {{{a}}_{{n}} - {{x}}_{{n}}^2 - {{y}}_{{n}}^2} \right]{{y}}_{{n}} + {\omega}_{{n}}{{x}}_{{n}} \\ & + {{G}}\mathop {\sum}\nolimits_{{{p}} = 1}^{{N}} {{{C}}_{{{np}}}\left( {{{y}}_{{p}}({{t}} - {\tau}) - {{y}}_{{p}}} \right)} + \beta _n{\eta}_n\left( {{t}} \right)\end{array}$$

where *G* denotes the global coupling weight, scaling equally the total input received in each brain area, and *τ* is a time lag. The SC matrix *C*_*np*_ is estimated and normalized from dMRI tractography (with a maximum of 0.2) and thus symmetric.

During optimization, the strength of connections in *C*_*np*_ is updated on the basis of the fit between the model output and the empirical NDTE flow matrix in terms of correlation. The empirical NDTE matrix is bidirectional and thus asymmetric. Hence, when updating the structural matrix, this will become asymmetric too. Thus, *C*_*np*_ is the GABIC.

We used a global optimization routine of MATLAB, namely tehe particle swarm optimizer. Particle swarm is a population-based algorithm, similar to the genetic algorithm, which has been widely used in treating ill-structured continuous, constrained as well as unconstrained function optimization problems^139–141^. A population of individuals (called particles) diffuse throughout the searching region of parameters, not dissimilar to swarming insects. At each step, the algorithm evaluates the objective function for each particle. In our case, the objective consisted of maximizing the correlation between the empirical and simulated NDTE matrix (that is, considering the causality between all pairs) by optimizing sequentially the noise level, *β*_*n*_, the time lag, *τ*, the local bifurcation parameters, *a*_*n*_, and most importantly the matrix *C*_*np*_. The initial values of noise were fixed thus: *β* = 0.02, *a*_*n*_ = −0.02, with the time lags also initialized to zero. The diffusion of the particles is optimally adapted by the algorithm to converge to a global maximum.

We remark that the whole-brain model generation of the NDTE matrix achieved here is a non-trivial problem, which is the result of using a non-linear model of a whole-brain coupling structure (SC) that does not correlate with the desired directional information flow (NDTE). This is essential for identifying the underlying GABIC matrix that can generate and mechanistically explain the emergence of the NDTE information flow—as well as other emergent properties such as FRIC.

An important caveat to the findings presented here is the seminal work by Judea Pearl in his book *Causality*^142^, where he shows that any framework of causal inference is based on inferring causal structures that are equivalent in terms of the probability distributions they generate; that is, they are indistinguishable from observational data, and could only be distinguished by manipulating the whole system.

Nevertheless, our NDTE framework—built from the probabilistic principles of mutual information as shown in the existing literature^25–27,109^—is well suited for our stated aim of determining the functional hierarchical organization of brain function by determining the directional causality.

In line with Pearl’s argument, we remark that the NDTE framework does capture the directionality of information flow needed for our purposes, but not the generative underlying physical coupling. For this we built the whole-brain GABIC model, which is able to capture the brain physical generators accounting for the causal influence of one neural system over another. We note that another promising framework for capturing the generators could be the dynamical causal modelling (DCM) framework^143^. However, it should be noted that DCM is currently limited to applications with very limited number of areas (at most approximately 10), although recent work has tried to apply the framework to the whole brain^144,145^.

### Reporting Summary

Further information on research design is available in the Nature Research Reporting Summary linked to this article.

## Supplementary information

Supplementary Information Supplementary Figs. 1 and 2.

Reporting Summary

Peer Review Information

## Data Availability

The multimodal neuroimaging data are freely available from HCP.

## Code availability

The code used to run the analysis from the HCP data is available on GitHub (https://github.com/decolab/nhb-ndte).
