# Supplementary material for: Revisiting the global workspace orchestrating the hierarchical organization of the human brain
Source: Nat Hum Behav. 2021 Jan 4;5(4):497–511. doi: 10.1038/s41562-020-01003-6 (PMC8060164; doi:10.1038/s41562-020-01003-6)
Supplement: Supplementary file 1 — Supplementary Figs. 1 and 2. [file 41562_2020_1003_MOESM1_ESM.pdf]

natureresearch

https://doi.org/10.1038/s41562-020-01003-6

Supplementary information

Revisiting the global workspace orchestrating the hierarchical organization of the human brain

In the format provided by the authors and unedited

Supplementary information

Revisiting the Global Workspace orchestrating the hierarchical organisation of the human brain

Gustavo Deco, Diego Vidaurre & Morten L. Kringelbach

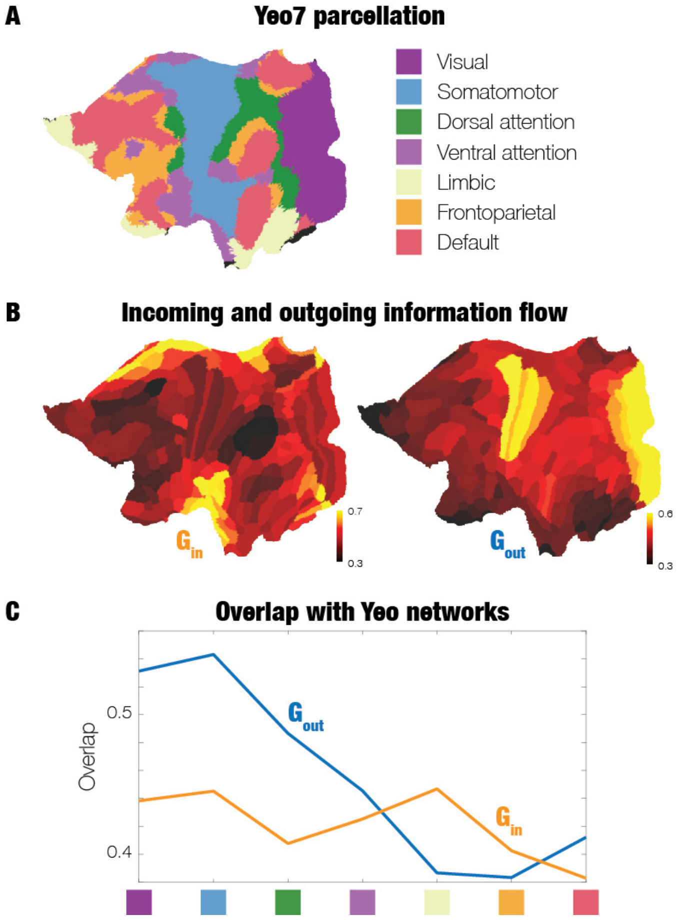

Supplemental Figure 1. Linking the incoming and outgoing information flow to the Yeo networks. A) *The panel shows the seven Yeo networks on a flatmap of the left hemisphere.* ***B)*** *The panel shows the flatmaps of the incoming,* G_{in}*, and outgoing,* G_{out}*, information flow in the Glasser parcellation.* ***C)*** *As can be seen visually, and quantified in the correlation plot,* G_{out} *most strongly overlaps with the sensory networks (visual and somatomotor) and much less with higher order networks (including the limbic and frontoparietal networks). On the other hand,* G_{in} *overlaps most strongly with the limbic network – and parts of the somatomotor network in the posterior insula.*

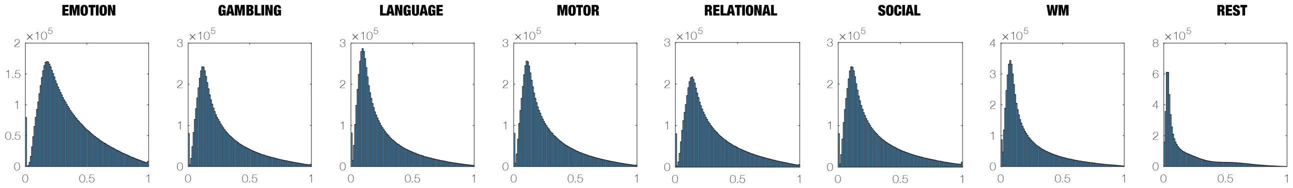

Supplemental Figure 2. Eight histograms of the p-values for the seven tasks and resting. *The figure shows all p-values for all pairs of timeseries across all participants in each of the eight conditions coming from the surrogates.*
