# Supplementary material for: Revisiting the global workspace orchestrating the hierarchical organization of the human brain
Source: Nat Hum Behav. 2021 Jan 4;5(4):497–511. doi: 10.1038/s41562-020-01003-6 (PMC8060164; doi:10.1038/s41562-020-01003-6)
Supplement: Supplementary file 2 — Reporting Summary [file 41562_2020_1003_MOESM2_ESM.pdf]

nature research

Corresponding author(s): Deco

Last updated by author(s): Sep 22, 2020

Reporting Summary

Nature Research wishes to improve the reproducibility of the work that we publish. This form provides structure for consistency and transparency in reporting. For further information on Nature Research policies, see our Editorial Policies and the Editorial Policy Checklist.

Statistics

For all statistical analyses, confirm that the following items are present in the figure legend, table legend, main text, or Methods section.

n/a	Confirmed	
☐	☒	The exact sample size (n) for each experimental group/condition, given as a discrete number and unit of measurement
☐	☒	A statement on whether measurements were taken from distinct samples or whether the same sample was measured repeatedly
☐	☒	The statistical test(s) used AND whether they are one- or two-sided<br>*Only common tests should be described solely by name; describe more complex techniques in the Methods section.*
☐	☒	A description of all covariates tested
☐	☒	A description of any assumptions or corrections, such as tests of normality and adjustment for multiple comparisons
☐	☒	A full description of the statistical parameters including central tendency (e.g. means) or other basic estimates (e.g. regression coefficient) AND variation (e.g. standard deviation) or associated estimates of uncertainty (e.g. confidence intervals)
☐	☒	For null hypothesis testing, the test statistic (e.g. F, t, r) with confidence intervals, effect sizes, degrees of freedom and P value noted<br>*Give P values as exact values whenever suitable.*
☒	☐	For Bayesian analysis, information on the choice of priors and Markov chain Monte Carlo settings
☒	☐	For hierarchical and complex designs, identification of the appropriate level for tests and full reporting of outcomes
☐	☒	Estimates of effect sizes (e.g. Cohen's d, Pearson's r), indicating how they were calculated

Our web collection on statistics for biologists contains articles on many of the points above.

Software and code

Policy information about availability of computer code

Data collection	Standard MRI and MEG scanner software
Data analysis	Standard imaging tools for preprocessing FSL (FMRIB Software Library), FreeSurfer, and the Connectome Workbench software.<br>Custom-made MATLAB scripts were used for whole-brain modelling, which are freely available

For manuscripts utilizing custom algorithms or software that are central to the research but not yet described in published literature, software must be made available to editors and reviewers. We strongly encourage code deposition in a community repository (e.g. GitHub). See the Nature Research guidelines for submitting code & software for further information.

Data

Policy information about availability of data

All manuscripts must include a data availability statement. This statement should provide the following information, where applicable:

- Accession codes, unique identifiers, or web links for publicly available datasets
- A list of figures that have associated raw data
- A description of any restrictions on data availability

The code is freely available from github and multimodal neuroimaging data from the experiment are available on HCP website

Field-specific reporting

Please select the one below that is the best fit for your research. If you are not sure, read the appropriate sections before making your selection.

☒ Life sciences ☐ Behavioural & social sciences ☐ Ecological, evolutionary & environmental sciences

For a reference copy of the document with all sections, see nature.com/documents/nr-reporting-summary-flat.pdf

Life sciences study design

All studies must disclose on these points even when the disclosure is negative.

Sample size	We used data of 1003 participants from the public available database from the Human Connectome Project (HCP) from the WU-Minn HCP Consortium.
Data exclusions	N/A
Replication	Findings from full dataset was replicated in 45 individuals with retest data
Randomization	N/A
Blinding	N/A

Reporting for specific materials, systems and methods

We require information from authors about some types of materials, experimental systems and methods used in many studies. Here, indicate whether each material, system or method listed is relevant to your study. If you are not sure if a list item applies to your research, read the appropriate section before selecting a response.

Materials & experimental systems

n/a	Involved in the study	
☒	☐	Antibodies
☒	☐	Eukaryotic cell lines
☒	☐	Palaeontology and archaeology
☒	☐	Animals and other organisms
☐	☒	Human research participants
☒	☐	Clinical data
☒	☐	Dual use research of concern

Methods

n/a	Involved in the study	
☒	☐	ChIP-seq
☒	☐	Flow cytometry
☐	☒	MRI-based neuroimaging

Human research participants

Policy information about studies involving human research participants

Population characteristics	We used data a group of 1003 participants from the public available database from the Human Connectome Project (HCP) from the WU-Minn HCP Consortium.
Recruitment	The data set used for this investigation was selected from the March 2017 public data release from the Human Connectome Project (HCP) where we chose the full sample
Ethics oversight	HCP

Note that full information on the approval of the study protocol must also be provided in the manuscript.

Magnetic resonance imaging

Experimental design

Design type	Resting state and task design
Design specifications	1003 HCP participants. The HCP website (http://www.humanconnectome.org/) provides the full details of participants, the acquisition and preprocessing of the data.
Behavioral performance measures	N/A

April 2020

Acquisition

Imaging type(s)	functional and diffusion MRI
Field strength	3T
Sequence & imaging parameters	The 1003 HCP participants were scanned on a 3-T connectome-Skyra scanner (Siemens). The HCP website (http://www.humanconnectome.org/) provides the full details of participants, the acquisition and preprocessing of the data.
Area of acquisition	Whole brain

Diffusion MRI ☒ Used ☐ Not used

Parameters: The HCP website (http://www.humanconnectome.org/) provides the full details of parameters for the dMRI data.

Preprocessing

Preprocessing software	Standardized methods using FSL (FMRIB Software Library), FreeSurfer, and the Connectome Workbench software
Normalization	FLIRT
Normalization template	CIFTI
Noise and artifact removal	The HCP website (http://www.humanconnectome.org/) provides the full details of the preprocessing of the data.
Volume censoring	As per HCP pipeline

Statistical modeling & inference

Model type and settings	Whole-brain model
Effect(s) tested	Spatiotemporal structure

Specify type of analysis: ☒ Whole brain ☐ ROI-based ☐ Both

Statistic type for inference (See Eklund et al. 2016)	N/A
Correction	N/A

Models & analysis

n/a	Involved in the study
☐	☒ Functional and/or effective connectivity
☒	☐ Graph analysis
☒	☐ Multivariate modeling or predictive analysis

Functional and/or effective connectivity: Pearson correlation
