# Supplementary material for: Revisiting the global workspace orchestrating the hierarchical organization of the human brain
Source: Nat Hum Behav. 2021 Jan 4;5(4):497–511. doi: 10.1038/s41562-020-01003-6 (PMC8060164; doi:10.1038/s41562-020-01003-6)
Supplement: Supplementary file 3 — Peer Review Information [file 41562_2020_1003_MOESM3_ESM.pdf]

natureresearch

# Peer Review Information

**Journal:** Nature Human Behaviour
**Manuscript Title:** Revisiting the Global Workspace orchestrating the hierarchical organisation of the human brain
**Corresponding author name(s):** Gustavo Deco & Morten Kringelbach

## Editorial Notes:

## Reviewer Comments & Decisions:

**Decision Letter, initial version:**

18th May 2020

Dear Gus,

Thank you once again for your manuscript, entitled "Revisiting the Global Workspace: Orchestration of the functional hierarchical organisation of the human brain", and for your patience during the peer review process.

Your Article has now been evaluated by 3 referees. You will see from their comments copied below that, although they find your work of potential interest, they have raised quite substantial concerns. In light of these comments, we cannot accept the manuscript for publication, but would be interested in considering a revised version if you are willing and able to fully address reviewer and editorial concerns.

We hope you will find the referees' comments useful as you decide how to proceed. If you wish to submit a substantially revised manuscript, please bear in mind that we will be reluctant to approach the referees again in the absence of major revisions. We are committed to providing a fair and constructive peer-review process. Do not hesitate to contact us if there are specific requests from the reviewers that you believe are technically impossible or unlikely to yield a meaningful outcome.

The reviews share one particular concern, which is the lack of evidence for the robustness and generalizability of your findings. In revision, we expect you to address the following important issues:

First, to address a robustness and validity concern, we expect a validation of the NDTE as a measure of directional functional connectivity.

Second, to demonstrate generalizability across tasks, we ask you to apply your analyses to all of the existing relevant tasks in the HCP; if for some reason the remaining tasks are not suitable, a new dataset that includes additional tasks should be used. Moreover, to demonstrate robustness, i.e. generalizability across datasets, we ask you to apply your analysis to the HCP re-test dataset, or ideally an entirely different dataset for external validation.

Third, we request that you use the DTI data from the HCP exhaustively instead of basing your analysis on the small subset of participants. Moreover, further reviewer requests for reanalysi that serve the purpose of improving the strength of evidence, such as the use of a fine-scale atlas, should also be met.

The reviewers suggest that in some instances, the previous literature could be better incorporated. We ask you to consider these recommendations, however, we do not mandate that you follow the reviewers' requests for specific citations.

Finally, your revised manuscript must comply fully with our editorial policies and formatting requirements. Failure to do so will result in your manuscript being returned to you, which will delay its consideration. To assist you in this process, I have attached a checklist that lists all of our requirements. I have also attached a template manuscript file that exemplifies our policies and formatting requirements. If you have any questions about any of our policies or formatting, please don't hesitate to contact me.

If you wish to submit a suitably revised manuscript we would hope to receive it within 6 months. We understand that the COVID-19 pandemic is causing significant disruptions which may prevent you from carrying out the additional work required for resubmission of your manuscript within this timeframe. If you are unable to submit your revised manuscript within 6 months, please let us know. We will be happy to extend the submission date to enable you to complete your work on the revision.

With your revision, please:

• Include a “Response to the editors and reviewers” document detailing, point-by-point, how you addressed each editor and referee comment. If no action was taken to address a point, you must provide a compelling argument. This response will be used by the editors to evaluate your revision and sent back to the reviewers along with the revised manuscript.

• Highlight all changes made to your manuscript or provide us with a version that tracks changes.

Please use the link below to submit your revised manuscript and related files:

***[REDACTED]***

<strong>Note:</strong> This URL links to your confidential home page and associated information about manuscripts you may have submitted, or that you are reviewing for us. If you wish to forward this email to co-authors, please delete the link to your homepage.

Thank you for the opportunity to review your work. Please do not hesitate to contact me if you have any questions or would like to discuss the required revisions further.

Best wishes
Marike

Anne-Marike Schiffer
Editor
Nature Human Behaviour

Reviewer expertise:

Reviewer #1: neural networks, connectome, DTI

Reviewer #2: neural networks, connectome, Psychiatry

Reviewer #3: neural networks, connectome, MEG

REVIEWER COMMENTS:

Reviewer #1:
Remarks to the Author:
The authors have conducted a thorough computational investigation with the HCP dataset to reveal the core regions (i.e., global workspace) and functional hierarchical organization of neuroinformation processing at the macroscopic level. The topic is critical for understanding brain function, and results seem intriguing. Here are some specific comments:

1. The key results are based on the NDTE framework. Regarding the directional functional connectivity, there have been other methods, like SEM and DCM. The validation for such estimations has been a big challenge, i.e., does the estimated direction really capture the direction of underlying information flow? The authors need to first validate this NDTE framework well.

2. The HCP have the re-test dataset. The authors can evaluate the reproducibility of the major results of resting fMRI. This can also provide further supports to the robustness of the global workspaces.

3. Whether the T1/T2 map can be used to reflect the myelin is very controversial. This should be discussed. In addition, how could the degree of myelin can be used as a proxy for anatomical hierarchical organization?

4. The spatial scale of this computational study is at the regional level. The implication of this macro-level findings to micro-level computational studies should be discussed. Particularly, despite of the two regional parcellation schemes, more discussion should be made for the influence of regional parcellation on the key findings.

Reviewer #2:
Remarks to the Author:

In this paper, the authors proposed to quantify he global workspace as the common regions across seven tasks and rest, in a common ‘functional rich club by determining the information flow between brain regions through a normalized directed transfer entropy framework. The causal robustness of this study was evaluated with lesioning a generative whole-brain model. The study is interesting. But a number of major issues needed to be addressed before it could be considered for publication.

1. The authors used the seven tasks from HCP datasets to derive the Global Workspace. However, the study doesn’t include all the task types, such as decision making. So the Global Workspace found out in this paper may be task-specific. That is, different task sets may result in different Global Workspace.

2. The six brain found in this study are in functional rich club. There is no clear evidence to relate this functional rich club to the Global Workspace. Moreover,the authors should compare the brain regions derived from the NDTE methods with the structural “rich club” that was derived from previous researches.

3. For the atlas used, you combined the fine-scale Glasser atlas and 18 subcortical regions. But the subcortical region is relatively coarser than cortical parcellation. It was suggested to verify the results on the Brainnetome atlas, which includes 210 cortical brain regions and 36 subcortical brain regions as a whole brain atlas (Fan L et al, Cerebral Cortex, 2016;26:508–3526). Besides, this paper derived the Global Workspace from the coarse DK80 atlas. It is not clear whether the Global Workspace derived from a coarse-scale atlas is the same as that derived from a fine-scale atlas.

4. In the Methods part, the authors mentioned that “From this large sample we further chose to replicate in the smaller subsample of 100 unrelated participants (54 females, 46 males, mean age = 29.1 +/- 3.7 years).”at line 410. But it did not found the corresponding results in the Results part. Moreover, the p values were defined for each NDTE in the Methods part, but there are no p-value analyses in the Results part.

5. The authors claimed that “FF is not as useful for establishing the full hierarchical organisation as the other NDTE flow measures.” in line 367, but there is no any evidence or a detailed the discussion for this conclusion.

6. For FRIC, the authors said “this quantitative framework can be used to identify the smallest core set of brain regions that integrate and orchestrate function in a given task” at line 105, while “running a simple algorithm that searches for the largest subset of regions” at line 600. Although here the “largest” means the subset with largest GFRIC, but it’s ambiguous.

7. There is no colorbar in Figure 2. Please also check other figures.

8. You use “GW” as the abbreviation for “Global Workspace” in line 1080, but “GNW” was used in line 1083. “GNW” and “GW” seem to be the same issue. Also in line 202 and 203.

9. In Figure 5, “Emp”, “FRIC”, and “nFRIC” should be defined in the legend.

10. In lines 160-161, the statistics should be given with both correlation coefficients and its corresponding p values. Also in other statistics.

11. From line 165 to 169, why not directly correlate the functional hierarchical organization between fMRI and MEG, when validate the results from fMRI?

12. “FF” in line 245 was not defined previously.

13. From line 147 to 149, the incoming and outgoing information flow should be compared quantitatively in detail using networks defined by Yeo.

14. The discussion from line 377-382 means the same thing as that from line 309 to 318.

15. The abbreviation for “HCP”, “NDTE”, and “FRIC” were defined for more than one time. They should be defined at the first time they appeared in the main article.

16. In line 456, a “,” was omitted before “but briefly”. Also in line 509, Equation (3), a “,” was omitted between yi+1 and yi. Besides, in equation (10), “=1” should be omitted in “i=1” to keep same format as other equations.

17. There in a parameter of “T” in Equation (4)-(7), but you did not mention how to choose this parameter.

18. The definitions for source and target in Equation (12) is opposite to those when defining Gin and Gout. It’s better to use uniform definition.

Reviewer #3:
Remarks to the Author:
This is a very interesting and novel paper on the functional core of regions that could potentially make up the global workspace. Topic is very relevant and analysis tools are state of the art and very novel. I have a couple of concerns and some open questions that need to be addressed, see below

If we consider the classical cognitive domains used for example in the field of cognitive neurology: learning and memory, social cognition, perceptual/motor/praxis, attention, language and executive functioning. I don’t think the included tasks actually cover all of these cognitive domains equally or sufficiently? For example, attention is always needed for every task, but not every task is suited to study attention modulation. If not, how certain are the authors that the intersection of the functional rich club involved in information flow for different tasks and rest make up the global workspace? How big is the chance the authors actually missed regions because of this limitation.

I suggest the authors to explain in the methods or maybe in the supplementary material in more detail the difference of the current NDTE approach to other ways to compute transfer entropy. The problem is that you usually need sufficient amount of data especially to compute the joint probabilities. Probabilities that are usually estimated using approaches such as binning or nearest neighbours. I guess the different definition based on the covariance matrices circumvents this. And if I am wrong here, please guide the reader familiar with other versions of TE how and why this NDTE is different to other versions of TE (apart from the normalisation that is already explained).

TE can modulate and be influenced by the frequencies and bandwidth of signals (see work from

(Lobier et al., 2014; Weber et al., 2017). Can the authors confirm that in and outflow are not an artefact of subtle differences in the frequencies and bandwidth of the different regions (for example FRIC vs non FRIC).

I cannot find any information on how vectors Xi or Yi were constructed. Did the authors apply some time delayed embedding (as discussed in book by (Wibral et al., 2014))? Or was it just the recent history up to a certain point i-T-1. How many windows were used for every subject? What was the influence and sensitivity of window width on TE estimation? See for example (Lobier et al., 2014) Fig 9. In this case for phase transfer entropy you need a minimum amount of data to get stable results. This is more easily achieved using electrophysiological recordings such as MEG/EEG, but not so easily with fMRI. I wonder how this would be for fMRI data, what is the cut off for the window width to achieve stable results with this method (if this is very different to previous methods using binning or nearest neighbours, please demonstrate and clarify in the supplements).

The authors claim that the circular time shift surrogate reconstruction preserves the whole statistical structure of the original timeseries. However, could the authors please clarify how this method preserves linear correlations when the time shift is different for two timeseries between which NDTE is computed. I am not able to see this, but please clarify if I am wrong here. Alternative would be Twin surrogates, a method that indeed preserves linear and nonlinear characteristics of the data (Romano et al., 2009).

I have an important concern regarding the Hopf model. The author introduce a novel way to induce directionality in the propagation of activity across nodes using empirical NDTE. However, I strongly believe that the reasoning is circular. Simulated NDTE based on connected oscillators informed with empirical NDTE matches the empirical data. This seems very obvious. The crux is to inform the model with updating/learning rules such that the empirical NDTE patterns becomes an emergent property. This would be the real challenge and I find this very important before I can regard these modelling results to be informative.

Optimisation of parameters indicates that an can either get bigger or smaller than 0, so operating in the steady state or limit cycle regime. Does an stays sufficiently close to the Hopf during the optimisation such that the noise can make the system switch between steady state and Hopf, or do we get nodes that are consistently within the steady state and some within the limit cycle regime? I doubt this would be realistic? In addition, how are these parameters actually optimised?

I understand the rationale for the Hopf model. Several neural mass models have shown to mimic functional connectivity when the operating point of the model was near a Hopf. Recent modelling work from Deco also demonstrated this for MEG functional connectivity data (however, please also mention recent work from (Tewarie et al., 2018) in the context of MEG and working points near Hopf). However, since the authors don not use biologically informed models, why not use models that explicitly model flow on a network, e.g. random walkers, diffusion models (Abdelnour et al., 2014)), epidemic spreading models. Even epidemic spreading models, or even wave equations on a network (see (Caputo et al., 2017)). Epidemic spreading models just beyond the critical epidemic threshold have also been useful to mimic directed connectivity (see (Meier et al., 2017)). This recent work demonstrated how direction of information flow (in terms of transfer entropy) in functional brain neworks emerges from local dynamics and structural topology. This previous work deserves to be discussed within the context of the modelling work here.

The authors state in the introduction: “For this purpose, we propose the concept of a ‘Functional Rich Club’ (FRIC) as the core set of regions, separate from the rest of the brain, which similar to a ‘club’ of functional hubs are characterized by a tendency to be more densely functionally connected among themselves than to other brain regions from where they receive integrative information”. However, I doubt that the FRIC metric that the authors have come up with actually quantify ‘this tendency to be more connected among themselves’. The set of brain regions with the highest amount of incoming directed flow does not necessarily have to be a set that it is densely connected among themselves. This clearly depends on the assortativity or assortative mixing in the network. If NDTE matrix Call is disassortative, then even if there is much inflow to the core set of regions this does not mean they are densely connected among themselves. The opposite would be true if there is strong assortative mixing (assortativity significantly higher than zero). Please see (Noldus and Van Mieghem, 2015) on assortativity for directed networks. I suggest the authors to analyse this before making claims about densely connected nodes.

In addition, you could hypothesize that in order to be a densely connected core, there should be significant in and out flow between rich club regions. Now, significant outflow has mainly been addressed within the context of bottom up processing from primary regions to higher order regions. But I am not sure the same reasoning holds for communication between ‘rich club regions’. The assumption made about FRIC naturally leads to putamen and nucleus accumbens as resulting nodes in the FRIC, since e.g. the putamen has a huge inflow and a rather limited out flow (just to the globus pallidus and substantia nigra). An alternative would be to compute some k-core characteristics of graphs, or perform some clustering analysis based on the link betweennes centrality. The latter would be less biased by the assumptions from the authors.
Related to this, how do the authors know that damaging FRIC nodes is really different than damaging other nodes. What if the authors simply carry out a targeted attack on the high indegree nodes in the network, does this give the same result as the FRIC vs non FRIC lesioning analysis of the authors. In other words, if another way of assessing ‘importance’ of nodes and lesioning of these nodes also give the same result then the following claim in the context of these modelling results would not hold: “This causally establishes the full FRIC network as having a key integrative role in orchestrating functional hierarchical organisation.” Or the authors have to show that these high indegree nodes are similar to FRIC nodes, but in that case, why would we need to compute the GFRIC metric anyway. Related to the GFRIC algorithm, how sure are the authors that their algorithm did not result in local maxima?

Please mention/discuss the work from (Hunt et al., 2016) in the context of myelination and connectivity as this work was the first who demonstrated a link between electrophysiological connectivity and myelination. Please mention the work from (Robinson, 2012) when mentioning the following in the methods: “two areas may be significantly correlated (in FC) although disconnected (EC=0) when strong indirect pathways connect them (i.e., large network effect).” As this was the first and most comprehensive work on indirect routes in relation to connectivity.

Why did the authors just used data from 32 subjects for DTI while fMRI data was obtained from 1000 subjects. I do not see the rationale for this huge discrepancy. The authors clearly state that we need huge and complete datasets to extract and obtain reliable results. When informing the Hopf model with a SC matrix and averaging out across more subjects their SC matrices would potentially also smear out false positive and negative links.

With regard to fMRI processing: Did the authors regress out average CSF and white matter signal

from the cortical fMRI timecourses. I only read about motion parameters, but can't see anything about CSF and white matter (see (Braun et al., 2012)).

With regard to MEG processing: How did you extract regional timecourses for MEG, was PCA used? Or did the authors compute an average across voxels or did they take the centroid voxel as representative voxel for an ROI. How was depth normalisation implemented? And how was source orientation estimated? I assume the authors used the provided head models from HCP? Did the authors use all three sessions? How did you correct/reduce signal leakage?

Please provide explicit mathematical description on how results from equation 8 eventually lead to Call.

Please make or enlarge Xlabels and ylabels Figure 2CF

Abdelnour, F., Voss, H.U., Raj, A., 2014. Network diffusion accurately models the relationship between structural and functional brain connectivity networks. Neuroimage 90, 335–347.
Braun, U., Plichta, M.M., Esslinger, C., Sauer, C., Haddad, L., Grimm, O., Mier, D., Mohnke, S., Heinz, A., Erk, S., 2012. Test–retest reliability of resting-state connectivity network characteristics using fMRI and graph theoretical measures. Neuroimage 59, 1404–1412.
Caputo, J.-G., Khames, I., Knippel, A., Panayotaros, P., 2017. Periodic orbits in nonlinear wave equations on networks. J. Phys. A Math. Theor. 50, 375101.
Hunt, B.A.E., Tewarie, P.K., Mougin, O.E., Geades, N., Jones, D.K., Singh, K.D., Morris, P.G., Gowland, P.A., Brookes, M.J., 2016. Relationships between cortical myeloarchitecture and electrophysiological networks. Proc. Natl. Acad. Sci. 113, 13510–13515.
Lobier, M., Siebenhühner, F., Palva, S., Palva, J.M., 2014. Phase transfer entropy: a novel phase-based measure for directed connectivity in networks coupled by oscillatory interactions. Neuroimage 85, 853–872.
Meier, J., Zhou, X., Hillebrand, A., Tewarie, P., Stam, C.J., Van Mieghem, P., 2017. The epidemic spreading model and the direction of information flow in brain networks. Neuroimage 152, 639–646.
Noldus, R., Van Mieghem, P., 2015. Assortativity in complex networks. J. Complex Networks 3, 507–542.
Robinson, P.A., 2012. Interrelating anatomical, effective, and functional brain connectivity using propagators and neural field theory. Phys. Rev. E 85, 11912.
Romano, M.C., Thiel, M., Kurths, J., Mergenthaler, K., Engbert, R., 2009. Hypothesis test for synchronization: twin surrogates revisited. Chaos An Interdiscip. J. Nonlinear Sci. 19, 15108.
Tewarie, P., Hunt, B.A.E., O'Neill, G.C., Byrne, A., Aquino, K., Bauer, M., Mullinger, K.J., Coombes, S., Brookes, M.J., 2018. Relationships between neuronal oscillatory amplitude and dynamic functional connectivity. Cereb. Cortex.
Weber, I., Florin, E., Von Papen, M., Timmermann, L., 2017. The influence of filtering and downsampling on the estimation of transfer entropy. PLoS One 12.
Wibral, M., Vicente, R., Lindner, M., 2014. Transfer entropy in neuroscience, in: Directed Information Measures in Neuroscience. Springer, pp. 3–36.

## Author Rebuttal to Initial comments

**Dear Marike**

**Further to our cover letter, below you will find the points raised in italics and our answers in BOLD.**

**Warm regards,**
**Gustavo and Morten**

*We hope you will find the referees' comments useful as you decide how to proceed. If you wish to submit a substantially revised manuscript, please bear in mind that we will be reluctant to approach the referees again in the absence of major revisions. We are committed to providing a fair and constructive peer-review process. Do not hesitate to contact us if there are specific requests from the reviewers that you believe are technically impossible or unlikely to yield a meaningful outcome.*

*The reviews share one particular concern, which is the lack of evidence for the robustness and generalizability of your findings. In revision, we expect you to address the following important issues:*

*First, to address a robustness and validity concern, we expect a validation of the NDTE as a measure of directional functional connectivity.*
**As you will see in our reply to reviewers below and in the revised ms, we have very carefully validated the NDTE framework and showed its robustness, whilst commenting on potential limitations to any causal framework.**

*Second, to demonstrate generalizability across tasks, we ask you to apply your analyses to all of the existing relevant tasks in the HCP; if for some reason the remaining tasks are not suitable, a new dataset that includes additional tasks should be used.*
**As we agreed over email, given that there are no other equally comprehensive neuroimaging dataset and that the strength of our analysis is that it includes the rest and task conditions in the same people, we have not included a completely different, incomplete dataset with different scanner parameters in a different population which would raise many more questions than answers. Below, in the answer to the reviewers, we have also pointed out the advantages of using the seven HCP tasks designed to cover the main aspects of human cognition in a scanner in a large group of individuals.**

*Moreover, to demonstrate robustness, i.e. generalizability across datasets, we ask you to apply your analysis to the HCP re-test dataset, or ideally an entirely different dataset for external validation.*
**We were very pleased to be able to convincingly demonstrate the robustness of our NDTE framework by following your suggestion and applying it to the HCP re-test dataset, which shows very high reproducibility (of up to a 0.97 correlation) despite containing the rest-**

**retest data from only a subsample of 45 participants of the 1003 participants (see new Figure S3).**

*Third, we request that you use the DTI data from the HCP exhaustively instead of basing your analysis on the small subset of participants.*
**We have computed the structural connectivity from the dMRI data from the full HCP cohort of 985 participants. As you will see in the revised ms, the structural connectivity is extremely similar to what we have obtained from the higher quality HCP data in 32 participants. If anything our original matrix would appear to be slightly better with more inter-hemispheric connections. This is not surprising given that the 32 participants were scanned for 89 minutes in the MGH scanner compared to 59 minutes for the standard HCP protocol (six runs of each approximately 9 minutes and 50 seconds).**

*Moreover, further reviewer requests for reanalysis that serve the purpose of improving the strength of evidence, such as the use of a fine-scale atlas, should also be met.*
**We have carried out the suggested reanalyses. This includes analysing everything with the suggested fine-scale Brainnetome atlas (246 cortical and subcortical regions).**

*The reviewers suggest that in some instances, the previous literature could be better incorporated. We ask you to consider these recommendations, however, we do not mandate that you follow the reviewers' requests for specific citations.*
**The reviewers' suggestions for further references to the existing literature were excellent and we have added all of them.**

***Reviewer #1:***
*Remarks to the Author:*
*The authors have conducted a thorough computational investigation with the HCP dataset to reveal the core regions (i.e., global workspace) and functional hierarchical organization of neuroinformation processing at the macroscopic level.*

**Thank you so much for your positive comment.**

*The topic is critical for understanding brain function, and results seem intriguing. Here are some specific comments:*

*1. The key results are based on the NDTE framework. Regarding the directional functional connectivity, there have been other methods, like SEM and DCM. The validation for such estimations has been a big challenge, i.e., does the estimated direction really capture the direction of underlying information flow? The authors need to first validate this NDTE framework well.*

**Thank you for posing this very relevant question. While it is certainly true that it is a big challenge to validate any measurement methods of directional information flow, we have worked hard to validate our framework and to cite and discuss the relevant literature on existing validations and potential limitations to any causal framework (including ours).**

**Our current framework was inspired by the work of Andrea Brovelli who used and validated the transfer entropy framework with neuroimaging data in his classic *Journal of Neuroscience* paper (Brovelli *et al.*, 2015). We added three elements to this powerful framework; namely normalisation, circular surrogates and aggregation of p-values to improve the reliability of the NDTE framework.**

**First, in order to show that our NDTE framework captures the main causal connectivity, we used the state-of-the-art framework and network proposed by Seth, Chorley and Barrett (Seth *et al.*, 2013). We simulate BOLD data using our generative Hopf model in the proposed network (see supplementary figure S9A). As shown in Figure S9C-D and detailed in the added Supplementary Methods and Results, our NDTE framework was able to identify the causal directionality of the underlying network (compare S9B with S9D). We note that for correct estimation, it is clearly important to use the two new key elements that we have added to the transfer entropy framework, namely circular surrogates and aggregation of p-values.**

**Second, in addition to the network validation that we provide, it is important to note that there have already been clear validations of the transfer entropy framework. Most importantly, it has already been shown that using surrogates is essential to avoid problems**

**with determining directionality (Quiroga *et al.*, 2000). It is well known that rather than measuring actual causality, directionality measures can reflect *intrinsic* properties of each of the signals. In particular, research has shown that directionality can even be reversed, which depends mainly on the noise level and the main frequencies of each of the signals. However, a solution to this problem was proposed by Quiroga (Quiroga *et al.*, 2002) who showed that such bias can easily be detected by using surrogates and that causality can be established using the time shifted surrogates that we use.**

**Third, another important validation is that the use of the time shift surrogates has been shown to give optimal results with several linear and nonlinear synchronisation measures (Pereda *et al.*, 2005; Quiroga *et al.*, 2002).**

**An important caveat is the seminal work by Judea Pearl, eloquently exposed in his book "Causality" (http://bayes.cs.ucla.edu/BOOK-2K/) (Pearl, 2009), where he shows that any framework of causal inference is based on inferring causal structures that are equivalent in terms of the probability distributions they generate; that is, they are indistinguishable from observational data, and could only be distinguished by manipulating the whole system. This limitation is true for any estimation method including SEM or DCM, however much they are validated, especially when it is known that there are many hidden variables, which makes the number of possible causal structures compatible with your data even larger.**

**Nevertheless, our NDTE framework – built from the probabilistic principles of mutual information – is perfectly suited for our stated aim of determining the functional hierarchical organisation of brain function by determining the directional causality. In line with Pearl's argument, we remark that the NDTE framework does capture the directionality of information flow needed for our purposes – but not the generative underlying physical coupling. For this we built the whole-brain GABIC model which is able to capture the brain physical generators accounting for the causal influence of one neural system over another.**

**Overall, as we have shown, thanks to the introduction of normalisation, circular surrogates and aggregation of p-values into our novel NDTE framework, our results are clearly meaningful and must reflect at least part of the underlying causal mechanisms. Furthermore, the validation of our methods shows that the NDTE framework is robust and highly reproducible.**

**In terms of direct comparison of the NDTE framework with DCM, it should be noted that DCM is currently limited to applications with very limited number of areas (at most approximately 10), although recent work has attempted to apply the framework to the whole-brain (Frassle *et al.*, 2017; Prando *et al.*, 2020).**

**We have added and expanded on all of this information on the important issue of validation in the appropriate places in the methods, results, discussion and references.**

*2. The HCP have the re-test dataset. The authors can evaluate the reproducibility of the major results of resting fMRI. This can also provide further supports to the robustness of the global workspaces.*

**We have run the proposed validation to generate the NDTE matrices (in the DK80 parcellation) for rest and the seven tasks in the re-test dataset and found very strong significant correlations between the original and the re-rest matrices (up to r=0.97 for rest). We have added this information to the results as well as a supplementary figure of the original and re-test NDTE matrices for all tasks and rest.**

*3. Whether the T1/T2 map can be used to reflect the myelin is very controversial. This should be discussed. In addition, how could the degree of myelin can be used as a proxy for anatomical hierarchical organization?*

**This is very true and we have added the following to the results:**

**"It should be noted that there is significant controversy in the literature as to whether the ratio of T1w and T2w images (T1w/T2w ratio) in the grey matter is a reliable estimate of cortical myelin content. Nevertheless, there is a growing consensus that T1w/T2w ratio is a marker of general white matter microstructure in both health and disease (Arshad *et al.*, 2017; Beer *et al.*, 2016; Righart *et al.*, 2017) provided that a correctly standardised measure of T1w/T2w is applied (Cooper *et al.*, 2019). Our analysis uses the neuroimaging in the HCP dataset of healthy participants is state-of-the-art with isotropic spatial resolution of 0.7mm, which enables highly accurate individual maps of cortical myelin content and thickness (Glasser *et al.*, 2016)."**

**In terms of using T1w/T2w for the hierarchical organisation we have added this to the discussion:**

**"Burt and colleagues (Burt *et al.*, 2018) remarked how the T1w/T2w ratio is a potential marker for hierarchy across species in that the ratio is high in primary sensory areas (visual, somatosensory and auditory) and the ratio is low in association areas. We therefore compared the NDTE framework to the group-averaged cortical T1w/T2w ratio as a potential non-invasive correlate for the static hierarchical position of a given brain region."**

*4. The spatial scale of this computational study is at the regional level. The implication of this macro-level findings to micro-level computational studies should be discussed. Particularly, despite of the two regional parcellation schemes, more discussion should be made for the influence of regional parcellation on the key findings.*

**We deliberately computed the results for two different well-established parcellations, a coarse parcellation, (DK80) and a fine parcellation (Glasser378) and for this revision we have added a medium parcellation (Brainnetome246) and coarse parcellation (Yeo7). The results on all these parcellations are very similar and consistent at the macroscale but there are subtle differences at the micro-level; which is not surprising since there is no optimal parcellation scheme in existence. We have added discussion on the influence of the spatial scale of the parcellation in the revised ms:**
**"As shown by our results here, the NDTE framework can be used for any spatial scale and yields very consistent results from the four parcellations we have used here (Yeo7, DK80, Brainnetome246 and Glasser360). Still, it is important to note that there is no current consensus about what is the right spatial parcellation scheme as shown by a recent paper by Eickhoff and colleagues reviewing the literature on the topographic organisation of the brain (Eickhoff *et al.*, 2018). Nevertheless it is remarkable how similar and consistent the results are at the macroscale, reflecting the functional organisation of the human brain at different spatial scales."**

**Reviewer #2:**
Remarks to the Author:
In this paper, the authors proposed to quantify he global workspace as the common regions across seven tasks and rest, in a common 'functional rich club by determining the information flow between brain regions through a normalized directed transfer entropy framework. The causal robustness of this study was evaluated with lesioning a generative whole-brain model. The study is interesting. But a number of major issues needed to be addressed before it could be considered for publication.
**Thank you for your positive and constructive advice; below we have addressed all the major (and minor) issues you have raised.**

1. The authors used the seven tasks from HCP datasets to derive the Global Workspace. However, the study doesn't include all the task types, such as decision making. So the Global Workspace found out in this paper may be task-specific. That is, different task sets may result in different Global Workspace.
**We understand your concern but there is currently no consensus in the field what constitutes the most comprehensive test of all cognitive task abilities. As outlined in the revised ms, we therefore chose to use the state-of-the-art neuroimaging of rest and tasks in the same participants with the same processing. Indeed, according to the HCP consortium the task battery was designed to cover as wide a range of neural systems as is feasible within realistic time constraints (see Barch et al. 2013). They write that "We assessed seven major domains that we think sample the diversity of neural systems that will be of interest to a wide range of individuals in the field, including: 1) visual, motion, somatosensory, and motor systems; 2) category specific representations; 3) working memory/cognitive control**

**systems; 4) language processing (semantic and phonological processing); 5) social cognition (Theory of Mind); 6) relational processing; and 7) emotion processing".**

**In the revised manuscript, we have added a thorough discussion of to what extent the seven HCP tasks cover the spectrum of full cognitive tasks and potential limitations. We raise the following points:**
*** We are using all of the seven tasks in the HCP dataset, which is the largest and most comprehensive dataset available from 1000 healthy participants. The tasks were designed to cover the full range of human cognitive abilities and for instance, the gambling task includes a decision-making component.**
*** To the best of our knowledge, there are no other equally comprehensive neuroimaging dataset that we could use. The strength of our analysis is that it includes the rest and task conditions in the SAME participants. Including a completely different, incomplete dataset with different scanner parameters in a different population would potentially raise many more questions than answers.**

2. The six brain regions found in this study are in functional rich club. There is no clear evidence to relate this functional rich club to the Global Workspace. Moreover, the authors should compare the brain regions derived from the NDTE methods with the structural "rich club" that was derived from previous researches.
**Thank you for your comment. The main motivation for defining what we call FRIC (functional rich club) is to determine the core set of regions, separate from the rest of the brain, which similar to a 'club' of functional hubs are characterized by a tendency to be more densely functionally connected among themselves than to other brain regions from where they receive integrative information. This notion is related to previous static descriptions of the *anatomical* rich club, which includes nodes in a network with a tendency for high-degree nodes to be more densely connected among themselves than nodes of a lower degree. However, unlike the anatomical rich club, FRIC is a dynamic measure based on bidirectional flow of information not constrained by anatomy and thus will change across different tasks.**

**Following Baars' original conception of global workspace, we propose to associate the Global Workspace with the intersection of the FRICs involved in resting state and a palette of cognitive tasks. There could, of course, be other definitions but at least this is a first functional quantification of Baars's concept based on functional neuroimaging data from a large set of participants. We have made this explicit in the ms.**

**With regards to the anatomical rich club, as defined by Van Heuvel and Sporns, following your suggestion, we computed this from our structural dMRI data and have described the findings in the main text along with a new supplementary figure comparing rich club to the intersection of the FRICs (our definition of global workspace). The two concepts are**

**complementary in nature since the rich club is purely anatomical and thus does not capture functional changes, ie it does not change between rest and different tasks. Nevertheless there is some overlap in the important regions of precuneus and isthmus cingulate cortex.**

**In the revised ms, we have added a supplementary figure and results:**
**"The proposed FRIC concept is functional in nature, thus changing with task and capturing the top regions involved in the relevant functional organisation of the task. This is different from the existing anatomical rich club concept (van den Heuvel and Sporns, 2011; Zamora-Lopez *et al.*, 2010), which is static in nature. Figure S6 shows the result of computing the anatomical rich club for the structural DK80 connectivity matrix and the comparison to the FRICs of the seven HCP tasks and their intersection in the Global Workspace. The results show that there are ten brain regions in the anatomical rich club: left and right precuneus, left and right thalamus, left and right superior frontal, left superior parietal, left insula and right isthmus cingulate cortices. Only three regions are shared with the Global Workspace, namely the precuneus (in both hemispheres) and the right isthmus cingulate cortex. Similarly, the FRIC of the individual tasks shows a similar overlap. This demonstrates the relevance of both concepts but highlights the dynamic nature of the FRIC concept, allowing for a more precise characterisation of the functional hierarchical organisation of the brain."**

3. For the atlas used, you combined the fine-scale Glasser atlas and 18 subcortical regions. But the subcortical region is relatively coarser than cortical parcellation. It was suggested to verify the results on the Brainnetome atlas, which includes 210 cortical brain regions and 36 subcortical brain regions as a whole brain atlas (Fan L et al, Cerebral Cortex, 2016;26:508–3526). Besides, this paper derived the Global Workspace from the coarse DK80 atlas. It is not clear whether the Global Workspace derived from a coarse-scale atlas is the same as that derived from a fine-scale atlas.
**Thank you for your suggestion. We have verified the results using the Brainnetome atlas and as you can see in the new supplementary Figure S4, the renderings of the NDTE matrices for rest and seven tasks are strongly consistent with our findings from DK80 and Glasser378.**

**With regards to computing the FRICs using the finer scale Brainnetome atlas, as you can appreciate from the rewritten methods in the ms, the computational complexity of our FRIC algorithm is NP hard. Nevertheless, we took considerable time to come up with an approximation of FRIC, as described in the revised Methods. Briefly, this method simply takes the intersection of the top $G_{in}$ regions across rest and seven tasks (and thus is only a rough approximation). However, the resulting global workspace is remarkably similar to the results of running the sophisticated and exhaustive algorithm (see new Figure S5).**

4. In the Methods part, the authors mentioned that “From this large sample we further chose to replicate in the smaller subsample of 100 unrelated participants (54 females, 46 males, mean age = 29.1 +/- 3.7 years).” at line 410. But it did not found the corresponding results in the Results part. Moreover, the p values were defined for each NDTE in the Methods part, but there are no p-value analyses in the Results part.
**Following your suggestions, everything is now run for the full sample. This is clarified in the text. We have also clarified that the p-values are computed for the NDTE matrices for all tasks and rest for every participant, which are then aggregated using Stouffer’s method and corrected using FDR. In a new Supplementary Figure S12, we now show the eight histograms of the p-values for all pairs of timeseries across all participants in the seven tasks and resting.**

5. The authors claimed that “FF is not as useful for establishing the full hierarchical organisation as the other NDTE flow measures.” in line 367, but there is no any evidence or a detailed the discussion for this conclusion.
**Thank you for this important point regarding the results in Supplementary Figure S8; we have expanded the discussion accordingly: “As shown in Figure S8, the FF hierarchy measure is similar to $G_{out}$ in that it is not discriminatory between the seven tasks. On the other hand it is only weakly correlated with $G_{in}$, the integrative measure of incoming information flow needed for establishing FRIC. This is to be expected given that FF hierarchy is an ordered measure of the fraction of feedforward and feedback connections, rather than a characterisation of a given brain region in terms of incoming and outgoing information flow to the rest of regions in the brain, which is what is needed to establish the meaningful functional hierarchical organisation. The results demonstrate that the FF hierarchy measure is not as useful for establishing the full hierarchical organisation compared with the proposed NDTE framework.”**

6. For FRIC, the authors said “this quantitative framework can be used to identify the smallest core set of brain regions that integrate and orchestrate function in a given task” at line 105, while “running a simple algorithm that searches for the largest subset of regions” at line 600. Although here the “largest” means the subset with largest GFRIC, but it’s ambiguous.
**We agree with you and have removed “smallest”.**

7. There is no colorbar in Figure 2. Please also check other figures.
**OK, done.**

8. You use “GW” as the abbreviation for “Global Workspace” in line 1080, but “GNW” was used in line 1083. “GNW” and “GW” seem to be the same issue. Also in line 202 and 203.
**OK, done**

9. In Figure 5, “Emp”, “FRIC”, and “nFRIC” should be defined in the legend.

**OK, done.**

10. In lines 160-161, the statistics should be given with both correlation coefficients and its corresponding p values. Also in other statistics.
**OK, done.**

11. From line 165 to 169, why not directly correlate the functional hierarchical organization between fMRI and MEG, when validate the results from fMRI?
**Ok, we have added a panel with the correlation between fMRI and MEG $G_{in}$ and $G_{out}$.**

12. "FF" in line 245 was not defined previously.
**OK, done.**

13. From line 147 to 149, the incoming and outgoing information flow should be compared quantitatively in detail using networks defined by Yeo.
**We have computed the overlap of the incoming and outgoing information flow with the Yeo networks, which is shown in Figure S11. We found that $G_{out}$ most strongly overlaps with sensory networks (visual and somatomotor) and much less with higher order networks (including the limbic and frontoparietal networks). On the other hand, $G_{in}$ overlap most strongly with the limbic network. As can be seen in the flatmap in the figure, there is also overlap with parts of the somatomotor network in the insula.**

14. The discussion from line 377-382 means the same thing as that from line 309 to 318.
**Yes, you are right. We have revised the ms accordingly.**

15. The abbreviation for "HCP", "NDTE", and "FRIC" were defined for more than one time. They should be defined at the first time they appeared in the main article.
**OK, done.**

16. In line 456, a "," was omitted before "but briefly". Also in line 509, Equation (3), a "," was omitted between yi+1 and yi. Besides, in equation (10), "=1" should be omitted in "i=1" to keep same format as other equations.
**Thank you! OK, done.**

17. There in a parameter of "T" in Equation (4)-(7), but you did not mention how to choose this parameter.
**We have added the criteria for choosing T:**
**"Following Kantz and Schreiber (Kantz and Schreiber, 1997), we estimated the autocorrelations of the empirical BOLD signals for all regions and all participants, and found that on average this decay to the first minimum corresponded to a value *T=10*."**

18. The definitions for source and target in Equation (12) is opposite to those when defining Gin and Gout. It's better to use uniform definition.
**Thank you, done.**

**Reviewer #3:**
Remarks to the Author:
This is a very interesting and novel paper on the functional core of regions that could potentially make up the global workspace. Topic is very relevant and analysis tools are state of the art and very novel.
**Thank you!**

I have a couple of concerns and some open questions that need to be addressed, see below

If we consider the classical cognitive domains used for example in the field of cognitive neurology: learning and memory, social cognition, perceptual/motor/praxis, attention, language and executive functioning. I don't think the included tasks actually cover all of these cognitive domains equally or sufficiently? For example, attention is always needed for every task, but not every task is suited to study attention modulation. If not, how certain are the authors that the intersection of the functional rich club involved in information flow for different tasks and rest make up the global workspace? How big is the chance the authors actually missed regions because of this limitation.
**Thank you for raising this important point, which was also raised by reviewer 2 in the first comment. As you will see in our extensive reply, there is currently no consensus in the field what constitutes the most comprehensive test of all cognitive task abilities. However, we have chosen to use the most comprehensive dataset available at this point which has the most tasks in the largest number of people with state-of-the-art neuroimaging with resting state data with a state-of-the-art preprocessing. Following your recommendation, we have added these points and the potential limitations of our approach to the discussion. In particular with regards to your point about whether the results have missed regions, we have added**
**"We note that even though we have used the most comprehensive dataset with the most participants and using tasks covering the full cognitive domain, in future it would be of considerable interest to compare the results in an even larger set of tasks and participants."**

I suggest the authors to explain in the methods or maybe in the supplementary material in more detail the difference of the current NDTE approach to other ways to compute transfer entropy. The problem is that you usually need sufficient amount of data especially to compute the joint probabilities. Probabilities that are usually estimated using approaches such as binning or nearest neighbours. I guess the different definition based on the covariance matrices circumvents this. And if I am wrong here, please guide the reader familiar with other versions of TE how and why

this NDTE is different to other versions of TE (apart from the normalisation that is already explained).

**Thank you for this important point. As you will see in our reply to reviewer 1, we have significantly expanded on our description of the NDTE framework, and how it relates to previous work and especially that of Brovelli and colleagues. We have fully validated the framework but following your comment, in addition we note in the Methods:**

**"The NDTE framework was inspired by the work of Brovelli and colleagues who used and validated a similar transfer entropy framework in neuroimaging data (Brovelli *et al.*, 2015). This framework uses a Gaussian approximation, ie only second order statistics of the involved entropies, which means, as shown below, that instead of estimating the probabilities, the method estimates the covariance which massively facilitates computation. Finally, as also outlined below, we add three key elements to this powerful framework; namely normalisation, circular surrogates and aggregation of p-values to improve the reliability and robustness of the NDTE framework."**

TE can modulate and be influenced by the frequencies and bandwidth of signals (see work from (Lobier et al., 2014; Weber et al., 2017). Can the authors confirm that in and outflow are not an artefact of subtle differences in the frequencies and bandwidth of the different regions (for example FRIC vs non FRIC).

**Thank you for this important remark. We checked the transfer entropy for 100 unrelated participants with and without our narrow bandfiltering and found that the NDTE matrices were very highly correlated (0.97, shown in new Figure S10). We have cited the MEG papers and note that this is not relevant for BOLD in the revised Methods:**

**"We note that it has been shown that transfer entropy can be modulated and influenced by the frequencies and bandwidth of the filter of high temporal resolution signals coming from MEG/EEG (Lobier *et al.*, 2014; Weber *et al.*, 2017), these potential obstacles related to the high temporal resolution are insignificant for the slow, narrowly filtered (0.01-0.2Hz) BOLD signal. We show that the NDTE DK80 matrices for the filtered and non-filtered version of resting state for 100 unrelated HCP participants correlate 0.97 (Figure S10)."**

I cannot find any information on how vectors Xi or Yi were constructed. Did the authors apply some time delayed embedding (as discussed in book by (Wibral et al., 2014))? Or was it just the recent history up to a certain point i-T-1. How many windows were used for every subject? What was the influence and sensitivity of window width on TE estimation? See for example (Lobier et al., 2014) Fig 9. In this case for phase transfer entropy you need a minimum amount of data to get stable results. This is more easily achieved using electrophysiological recordings such as MEG/EEG, but not so easily with fMRI. I wonder how this would be for fMRI data, what is the cut off for the window width to achieve stable results with this method (if this is very different to previous methods using binning or nearest neighbours, please demonstrate and clarify in the supplements).

**Thank you for spotting the missing information. In line with our reply to Reviewer 2, point 17, we have now clarified as follows: "... indicates the whole activity level of the past of X (filtered BOLD signal sampled in TR) in a time window of length T up to and including the time point i (i.e. Xi=[Xi Xi-1 … Xi-(T-1)]). Following Kantz and Schreiber (Kantz and Schreiber, 1997), we estimated the autocorrelations of the empirical BOLD signals for all regions and all participants, and found that on average this decay to the first minimum corresponded to a value T=10."**

The authors claim that the circular time shift surrogate reconstruction preserves the whole statistical structure of the original timeseries. However, could the authors please clarify how this method preserves linear correlations when the time shift is different for two timeseries between which NDTE is computed. I am not able to see this, but please clarify if I am wrong here. Alternative would be Twin surrogates, a method that indeed preserves linear and nonlinear characteristics of the data (Romano et al., 2009).
**This is an excellent point which has been addressed in detail by the research of Rodrigo Quian Quiroga. There have been many suggestions of how best to construct surrogate data, e.g (Pereda *et al.*, 2005) and Appendix A in particular. A first approach is to construct time series with the same power spectrum (i.e. autocorrelation function) of the original ones, but with chance crosscorrelation. A more refined approach is to construct surrogates that also maintain the amplitude distribution (AAFT) (Schreiber and Schmitz, 1996). However, this is based on an iterative procedure that does not always converge. In contrast, in (Quiroga *et al.*, 2002) a very simple solution was proposed: to shift one dataset with respect to the other. This way the properties of each signal (amplitude and autocorrelation spectrum, etc) are maintained because the signals are exactly the same, but the cross-correlation is reduced to chance levels - which is exactly what is required, as the hypothesis testing is whether a synchronisation or directionality value is larger than chance. Furthermore, the use of the time shift surrogates were already shown to give optimal results with several linear and nonlinear synchronisation measures (Pereda *et al.*, 2005; Quiroga *et al.*, 2002). We have added this relevant information in the Methods.**

I have an important concern regarding the Hopf model. The authors introduce a novel way to induce directionality in the propagation of activity across nodes using empirical NDTE. However, I strongly believe that the reasoning is circular. Simulated NDTE based on connected oscillators informed with empirical NDTE matches the empirical data. This seems very obvious. The crux is to inform the model with updating/learning rules such that the empirical NDTE patterns become an emergent property. This would be the real challenge and I find this very important before I can regard these modelling results to be informative.
**Thank you for raising this concern. We have improved the methods to clear the potential misunderstanding of circularity and stress the difficulty of creating a generative model of whole-brain directional information flow. This is the first time this has been achieved - and could have been a paper in its own right.**

**In the last two decades of whole-brain modelling, we and others have modelled the empirical data in terms of static FC measures initially (Deco *et al.*, 2014; Honey *et al.*, 2007) - and lately dynamics FCD measurements (Deco *et al.*, 2017). This is achieved by optimising a single global coupling parameter such that the empirical and simulated measures are as similar as possible. The results have been very informative for understanding the underlying mechanisms of brain function, see for example reviews (Breakspear, 2017; Deco and Kringelbach, 2014; Deco *et al.*, 2015).**

**However, until now, there has not been a generative model that can explain the bidirectional flow of information across the whole-brain, which is non-trivial given the structural connectivity and NDTE matrices are not strongly correlated. The solution found here is achieved by using a particle swarm non-linear global optimisation method to optimise the generative conductivities of the existing anatomical fibres, here called "Generative Anatomically-constrained BIdirectional Connectivity" (GABIC). In terms of complexity, this is very different from our usual exhaustive linear optimisation of one global coupling parameter.**

**The resulting model reported here is very important for the results since it allowed us to study 'emergent properties' such as the causal relevance of the FRIC for brain dynamics which would be impossible without a mechanistic generative model.**

**In terms of your concern with circularity, we note that in a circular argument, 'circulus in probando', an argument is said to be logically valid since if the premises are true, the conclusion must be true. This, we would argue, is different from what we have achieved. Here, we have not created a model that re-creates the NDTE matrix (so the conclusion does not replicate the premise). Instead, we find the GABIC matrix that *can generate* and mechanistically *explain* the emergence of the NDTE information flow – as well as other emergent properties such as FRIC. Moreover, note that the whole-brain model generation of the NDTE matrix is a non-trivial, and is the result of using a non-linear coupled model of a coupling structure (SC) that does not correlate with the desired directional information flow (NDTE). We have added these clarifications to the revised ms.**

Optimisation of parameters indicates that a_n can either get bigger or smaller than 0, so operating in the steady state or limit cycle regime. Does a_n stays sufficiently close to the Hopf during the optimisation such that the noise can make the system switch between steady state and Hopf, or do we get nodes that are consistently within the steady state and some within the limit cycle regime? I doubt this would be realistic?

**This is an interesting point, which we have addressed exhaustively in the past, for example in the original whole-brain modelling Hopf paper (Deco *et al.*, 2017) and for example in the whole-brain modelling paper of DBS in PD (Saenger *et al.*, 2017). In brief, in our first paper establishing the Hopf model, Figure 5 shows the derivation of the dynamical core, which we call the vector of the bifurcation parameters, $a_n$, in all regions, n. It has been shown that the optimised $a_n$ forms a distribution of negative and positive values, consistent with the hetegeneous mixture of regions at bifurcation, steady state or limit cycle, arising**

**from the optimisation of the power spectrum of the regions (Deco *et al.*, 2017; Saenger *et al.*, 2017). Thus the proposed model is highly realistic by fitting important aspect of the empirical data including the power spectrum. Even more, in the modelling paper of DBS ON-OFF in PD measured in fMRI, we show broad distributions of $a_n$ for stimulation ON and OFF in PD and for healthy, age-matched controls. We have clarified this in the Methods of the revised ms.**

In addition, how are these parameters actually optimised?
**Thank you, in the revised ms, we have further clarified that we use the Particle swarm algorithm, which updates the noise level, $\beta_n$, the time lag, τ, the local bifurcation parameters, $a_n$, and most importantly the matrix $C_{np}$.**

I understand the rationale for the Hopf model. Several neural mass models have shown to mimic functional connectivity when the operating point of the model was near a Hopf. Recent modelling work from Deco also demonstrated this for MEG functional connectivity data (however, please also mention recent work from (Tewarie et al., 2018) in the context of MEG and working points near Hopf). However, since the authors don not use biologically informed models, why not use models that explicitly model flow on a network, e.g. random walkers, diffusion models (Abdelnour et al., 2014)), epidemic spreading models. Even epidemic spreading models, or even wave equations on a network (see (Caputo et al., 2017)). Epidemic spreading models just beyond the critical epidemic threshold have also been useful to mimic directed connectivity (see (Meier et al., 2017)). This recent work demonstrated how direction of information flow (in terms of transfer entropy) in functional brain networks emerges from local dynamics and structural topology. This previous work deserves to be discussed within the context of the modelling work here.
**We agree, and have expanded the Discussion to reference these important papers. We have also noted that we chose the Stuart-Landau Hopf model since this has been shown that this is the most universal non-linear oscillator, which has been applied broadly in a large set of completely different physical systems (Kuramoto, 1984) including neuroscience.**

The authors state in the introduction: "For this purpose, we propose the concept of a 'Functional Rich Club' (FRIC) as the core set of regions, separate from the rest of the brain, which similar to a 'club' of functional hubs are characterized by a tendency to be more densely functionally connected among themselves than to other brain regions from where they receive integrative information". However, I doubt that the FRIC metric that the authors have come up with actually quantify 'this tendency to be more connected among themselves'. The set of brain regions with the highest amount of incoming directed flow does not necessarily have to be a set that it is densely connected among themselves. This clearly depends on the assortativity or assortative mixing in the network. If NDTE matrix Call is disassortative, then even if there is much inflow to the core set of regions this does not mean they are densely connected among themselves. The opposite would be true if there is strong assortative mixing (assortativity significantly higher

than zero). Please see (Noldus and Van Mieghem, 2015) on assortativity for directed networks. I suggest the authors to analyse this before making claims about densely connected nodes.

**Many thanks for raising these important issues, which made us realise that we need to better clarify how the FRIC algorithm works, in particular how this was rigorously statistically tested. As you can now see in the revised Methods, the FRIC algorithm iteratively expands the FRIC by adding a new node to the existing club until the FRIC value of the FRIC club is no longer statistically significant. The FRIC algorithm tests if a FRIC has a significantly larger FRIC value than the all other possible surrogate clubs. The FRIC value is computed as the sum of the connections between all club members plus the sum of all the incoming connections for the club members minus the sum of all outgoing connections for the club members, ie as shown in equation 11: $\sum_k C_{All_k} + \sum_k G_{in}(k) - \sum_k G_{out}(k)$. This FRIC value is also used computed for all the permutations of all other clubs that can be made from the same number of existing nodes (with length k). Given the challenges of the combinatorics, we used a Monte Carlo simulation to generate the most similar and challenging permutations of the FRIC, ie where comparing a potential new FRIC club of size k, the surrogate clubs (with k members) have the same k-1 members with an added random new member. We run this procedure iteratively, starting from the top incoming $G_{in}$ regions, and continue while p-value of the comparison between the FRIC club and surrogates is smaller than 0.05.**

**In this sense, this clarifies that FRIC is computing exactly our definition of global workspace, and not a graph theoretical rich club at the functional level.**

**We were interested in your idea regarding assortativity and consulted seminal rich club paper of Van den Heuvel and Sporns, where they show that their rich club networks have a low assortativity (see Table 1). Furthermore in the excellent book “Fundamentals of Brain Network Analysis” by Fornito, Zalesky and Bullmore, on page 204, they state: “It is easy to see how rich-club organization can be confused with assortativity: the rich-club coefficient measures the density of connectivity between high-degree nodes, whereas assortativity measured the tendency of nodes with similar degree to connect to each other. The difference between these measures is that assortativity, when specified in terms of degree, describes a relationship between nodes across all levels of i. The rich-club coefficient is only concerned with connectivity amongst nodes with degree greater than k and makes no statement about the connectivity of nodes with lower degree. Another way of understanding this distinction between assortativity and the rich-club coefficient is to think about how these measures relate to Borgatti and Everett's (2000) model of a network with strong core-periphery structure (Figure 6.9). In this model, core nodes are strongly connected to each other and to peripheral nodes. The peripheral nodes, in turn, are only weakly connected amongst themselves. This organization is consistent with a rich-club, to the extent that the core will comprise the highest degree nodes in the network, and these nodes will also be densely connected with each other. However, it is not characteristic of a network with high assortativity, since an assortative network will have strong connectivity between high-degree nodes, strong connectivity between low-degree nodes, and only weak**

**connectivity between high-degree and low-degree nodes. In fact, the model depicted in the left panel of Figure 6.9 is consistent with a disassortative organization, since hubs are strongly connected with low-degree nodes, which in turn are not connected with each other." (Fornito *et al.*, 2016).**

**We have added some of these important points to the discussion, thank you. Finally, as you can see in our reply to Reviewer 2, we have computed the rich club for the structural connectivity and compared this directly to the FRIC in the results, a new Supplemental Figure S6 and in the discussion.**

In addition, you could hypothesize that in order to be a densely connected core, there should be significant in and out flow between rich club regions. Now, significant outflow has mainly been addressed within the context of bottom up processing from primary regions to higher order regions. But I am not sure the same reasoning holds for communication between 'rich club regions'. The assumption made about FRIC naturally leads to putamen and nucleus accumbens as resulting nodes in the FRIC, since e.g. the putamen has a huge inflow and a rather limited out flow (just to the globus pallidus and substantia nigra). An alternative would be to compute some k-core characteristics of graphs, or perform some clustering analysis based on the link betweenness centrality. The latter would be less biased by the assumptions from the authors.
**Thank you for this suggestion. We have clarified above how we are in fact checking if there is significant flow between FRIC regions (compared to all other permutations of possible clubs). This leads to the inclusion of some subcortical regions like the nucleus accumbens and the putamen. While there could of course other ways of defining FRIC, in the revised version we have shown that our method is robust. Still, we have added a caveat suggesting that potential improvement to FRIC could be an important avenue for future research.**

Related to this, how do the authors know that damaging FRIC nodes is really different than damaging other nodes. What if the authors simply carry out a targeted attack on the high indegree nodes in the network, does this give the same result as the FRIC vs non FRIC lesioning analysis of the authors. In other words, if another way of assessing 'importance' of nodes and lesioning of these nodes also give the same result then the following claim in the context of these modelling results would not hold: "This causally establishes the full FRIC network as having a key integrative role in orchestrating functional hierarchical organisation." Or the authors have to show that these high in-degree nodes are similar to FRIC nodes, but in that case, why would we need to compute the GFRIC metric anyway.
**Thank you for this important question which we have directly addressed in the revised ms by recomputing the model lesioning by adding a third comparison biased as you suggested. We have made a new Figure 5 with a revised figure legend and describe the results as follows:**
**"Figure 5B shows the result of lesioning all FRIC compared to non-FRIC members in the whole-brain model, which causes very significant breakdown in information flow. When measuring this in terms of correlation between empirical and simulated NDTE matrices we**

**found the relevant comparisons showing that there is a highly significant change in the fitting of the model when FRIC members are lesioned compared to the lesioning of non-FRIC members selected from all other regions ($p<10^{-30}$, compare second and third columns in Figure 5B). In order to make the comparison even more challenging, we biased the selection of the non-FRIC regions to only include the top 50% of the $G_{in}$ regions ($p<10^{-30}$, compare second and fourth columns in Figure 5B). Furthermore, in Figure 5C, we show the same relevant comparisons for measuring the significant consequences of detecting significant differences in the number of FRIC members detected when lesioning the original empirical FRIC members compared to empirical non-FRIC members randomly selected ($p<10^{-30}$, compare second and fourth columns in Figure 5C) and selected from top 50% of $G_{in}$ regions ($p<10^{-30}$, compare second and fourth columns in Figure 5C). This is strong causal evidence for the major significance of FRIC in orchestrating functional organisation."**

Related to the GFRIC algorithm, how sure are the authors that their algorithm did not result in local maxima?
**Like with all global optimisation methods, the particle swarm optimization algorithm cannot guarantee to have found the global maximum for a particular problem. Still, as we have clarified in the revised ms, we chose this particular optimisation algorithm since it has been widely used in treating ill-structured continuous, constrained as well as unconstrained function optimization problems (Del Valle *et al.*, 2008; Kennedy and Eberhart, 1995; Sengupta *et al.*, 2019).**

Please mention/discuss the work from (Hunt et al., 2016) in the context of myelination and connectivity as this work was the first who demonstrated a link between electrophysiological connectivity and myelination.
**Thank you, we have added this important information to the revised ms.**

Please mention the work from (Robinson, 2012) when mentioning the following in the methods: "two areas may be significantly correlated (in FC) although disconnected (EC=0) when strong indirect pathways connect them (i.e., large network effect)." As this was the first and most comprehensive work on indirect routes in relation to connectivity.
**Yes, you are right, we have added this to the revised ms.**

Why did the authors just used data from 32 subjects for DTI while fMRI data was obtained from 1000 subjects. I do not see the rationale for this huge discrepancy. The authors clearly state that we need huge and complete datasets to extract and obtain reliable results. When informing the Hopf model with a SC matrix and averaging out across more subjects their SC matrices would potentially also smear out false positive and negative links.
**Thank you for this excellent question. We used the HCP dataset from 32 subjects because the 89 minutes of dMRI scanning for each participants is of the highest quality and give**

**excellent results for tractography. We have followed your suggestion and recomputed the structural connectivity matrix from the dMRI data obtained using the standard HCP parameters for all usable 985 HCP participants, which takes a shorter period of 59 minutes. We had opted to use the special dMRI data from the 32 participants given that this is optimised to give the best possible data registered the HCP template. As you can see in Figure S7 in the revised ms, the structural connectivity matrix for this is extremely similar to what we have obtained from the higher quality HCP data. If anything our original matrix is slightly better with more interhemispheric connections. As you can see in the results section of the revised ms, we recomputed the whole-brain Hopf model of the empirical NDTE and the resulting FRIC with both SC matrices. Comparing the results between the model NDTEs obtained using very computer intensive particle swarm algorithm for both the standard and special SC matrices give identical results.**

With regard to fMRI processing: Did the authors regress out average CSF and white matter signal from the cortical fMRI timecourses. I only read about motion parameters, but can't see anything about CSF and white matter (see (Braun et al., 2012)).
**With regards to the fMRI preprocessing, we have made it clearer in the revised ms that we used the standard HCP pipeline which does not regress out CSF and uses FIX to remove artefacts, see (Glasser *et al.*, 2016).**

With regard to MEG processing: How did you extract regional timecourses for MEG, was PCA used? Or did the authors compute an average across voxels or did they take the centroid voxel as representative voxel for an ROI. How was depth normalisation implemented? And how was source orientation estimated? I assume the authors used the provided head models from HCP? Did the authors use all three sessions? How did you correct/reduce signal leakage?
**Thank you for these important questions. Please note that we only included the MEG data to demonstrate in principle that NDTE can be applied to other modalities - but that this is NOT the main part of the paper but rather an exciting avenue for future research. We agree that the description of the MEG preprocessing could be improved and have done this in the revised ms in the following way:**

**"For each participant, the MEG data were acquired in a single continuous run comprising resting state. As a starting point we used the preprocessed MEG data from the HCP database. At this level of preprocessing, removal of artefactual independent components, bad samples and channels has already been performed. Following the preprocessing pipeline detailed in (Vidaurre *et al.*, 2018), MEG data were then downsampled to 250Hz using an anti-aliasing filter, filtered out frequencies below 1Hz, performed the co-registration with the head models provided by the HCP, and source-reconstructed using LCMV beamforming (Hillebrand and Barnes, 2005) to the 62 cortical regions of the DK80 parcellation. For each region, a single time series was computed as the first principal component of the voxels within that parcel. Each region's time series was then**

**standardized to have mean equal zero and standard deviation equal one, so that the amount of variance was always the same regardless of the depth of the region. In order to project the results to brain space, we used a weighted mask, where each region had its maximum value at the center of gravity."**

**With regards to your question about signal leakage, this is, as you will know, an on-going important research question. We agree that this can be an important preprocessing step, but given the size of the parcellation, we decided not to apply leakage correction in this case. The main argument is that the amount of signal removal for the considered number of cortical regions (62) was too large for any subsequent analysis. Some new methods may using a "softer" version of the existing leakage correction techniques could potentially be useful, but devising this falls out of the scope of the present work. Overall, the impact of potential leakage correction on our results is likely to be minor, given the similarity to the fMRI results. Still, we have now flagged this as a limitation of the study within the manuscript:**

**"We did not apply correction for spatial leakage (volume conduction), because the amount of signal removal would have been too large given the number of regions in the parcellation, therefore making the rest of the analysis not possible. Although the similarity with the fMRI results suggests that the impact of signal leakage is not too disruptive for the conclusions of this study, this remains a limitation of this work, and future efforts will be dedicated to rigorously assess the impact of the different approaches to leakage correction on the NDTE metric, as well as to consider "softer" versions of such leakage correction approaches that can be used in this case."**

Please provide explicit mathematical description on how results from equation 8 eventually lead to C_all.

**Thank you for this excellent comment. We changed the notation to make it clear and mathematically consistent in the Methods of the revised ms.**

Please make or enlarge Xlabels and ylabels Figure 2CF

**OK, done.**

**References**

Arshad, M., Stanley, J. A. and Raz, N. (2017) Test-retest reliability and concurrent validity of in vivo myelin content indices: Myelin water fraction and calibrated T1 w/T2 w image ratio. *Human brain mapping* **38,** 1780-1790.

Beer, A., Biberacher, V., Schmidt, P., Righart, R., Buck, D., Berthele, A., Kirschke, J., Zimmer, C., Hemmer, B. and Muhlau, M. (2016) Tissue damage within normal appearing white matter in early multiple sclerosis: assessment by the ratio of T1- and T2-weighted MR image intensity. *Journal of neurology* **263,** 1495-1502.

Breakspear, M. (2017) Dynamic models of large-scale brain activity. *Nature neuroscience* **20,** 340-352.
Brovelli, A., Chicharro, D., Badier, J. M., Wang, H. and Jirsa, V. (2015) Characterization of Cortical Networks and Corticocortical Functional Connectivity Mediating Arbitrary Visuomotor Mapping. *The Journal of neuroscience : the official journal of the Society for Neuroscience* **35,** 12643-12658.
Burt, J. B., Demirtas, M., Eckner, W. J., Navejar, N. M., Ji, J. L., Martin, W. J., Bernacchia, A., Anticevic, A. and Murray, J. D. (2018) Hierarchy of transcriptomic specialization across human cortex captured by structural neuroimaging topography. *Nature neuroscience* **21,** 1251-1259.
Cooper, G., Finke, C., Chien, C., Brandt, A. U., Asseyer, S., Ruprecht, K., Bellmann-Strobl, J., Paul, F. and Scheel, M. (2019) Standardization of T1w/T2w Ratio Improves Detection of Tissue Damage in Multiple Sclerosis. *Frontiers in neurology* **10,** 334.
Deco, G. and Kringelbach, M. L. (2014) Great Expectations: Using Whole-Brain Computational Connectomics for Understanding Neuropsychiatric Disorders. *Neuron* **84,** 892-905.
Deco, G., Kringelbach, M. L., Jirsa, V. and Ritter, P. (2017) The Dynamics of Resting Fluctuations in the Brain: Metastability and its Dynamical Core [bioRxiv 065284]. *Scientific Reports* **7,** 3095.
Deco, G., Ponce-Alvarez, A., Hagmann, P., Romani, G. L., Mantini, D. and Corbetta, M. (2014) How local excitation-inhibition ratio impacts the whole brain dynamics. *The Journal of neuroscience : the official journal of the Society for Neuroscience* **34,** 7886-7898.
Deco, G., Tononi, G., Boly, M. and Kringelbach, M. L. (2015) Rethinking segregation and integration: contributions of whole-brain modelling *Nat Rev Neurosci* **16,** 430-439.
Del Valle, Y., Venayagamoorthy, G. K., Mohagheghi, S., Hernandez, J.-C. and Harley, R. G. (2008) Particle swarm optimization: basic concepts, variants and applications in power systems. *IEEE Transactions on evolutionary computation* **12,** 171-195.
Eickhoff, S. B., Constable, R. T. and Yeo, B. T. T. (2018) Topographic organization of the cerebral cortex and brain cartography. *NeuroImage* **170,** 332-347.
Fornito, A., Zalesky, A. and Bullmore, E. (2016) *Fundamentals of Brain Network Analysis*. Academic Press: New York.
Frassle, S., Lomakina, E. I., Razi, A., Friston, K. J., Buhmann, J. M. and Stephan, K. E. (2017) Regression DCM for fMRI. *NeuroImage* **155,** 406-421.
Glasser, M. F., Smith, S. M., Marcus, D. S., Andersson, J. L., Auerbach, E. J., Behrens, T. E., Coalson, T. S., Harms, M. P., Jenkinson, M., Moeller, S., Robinson, E. C., Sotiropoulos, S. N., Xu, J., Yacoub, E., Ugurbil, K. and Van Essen, D. C. (2016) The Human Connectome Project's neuroimaging approach. *Nature neuroscience* **19,** 1175-1187.
Hillebrand, A. and Barnes, G. R. (2005) Beamformer analysis of MEG data. *Int Rev Neurobiol* **68,** 149-171.
Honey, C. J., Kötter, R., Breakspear, M. and Sporns, O. (2007) Network structure of cerebral cortex shapes functional connectivity on multiple time scales. *Proceedings of the National Academy of Sciences of the United States of America* **104,** 10240-10245.

Kantz, H. and Schreiber, T. (1997) *Nonlinear time series analysis*. Cambridge University Press: Cambridge.

Kennedy, J. and Eberhart, R. (1995) Particle Swarm Optimization. *Proceedings of the IEEE International Conference on Neural Networks* **4,** 1942-1948.

Kuramoto, Y. (1984) *Chemical Oscillations,Waves, and Turbulence*. Springer-Verlag: Berlin.

Lobier, M., Siebenhuhner, F., Palva, S. and Palva, J. M. (2014) Phase transfer entropy: a novel phase-based measure for directed connectivity in networks coupled by oscillatory interactions. *NeuroImage* **85 Pt 2,** 853-872.

Pearl, J. (2009) *Causality. Models, reasoning and inference*. Cambridge University Press: Cambridge.

Pereda, E., Quiroga, R. Q. and Bhattacharya, J. (2005) Nonlinear multivariate analysis of neurophysiological signals. *Prog Neurobiol* **77,** 1-37.

Prando, G., Zorzi, M., Bertoldo, A., Corbetta, M., Zorzi, M. and Chiuso, A. (2020) Sparse DCM for whole-brain effective connectivity from resting-state fMRI data. *NeuroImage* **208,** 116367.

Quiroga, R. Q., Arnhold, J. and Grassberger, P. (2000) Learning driver-response relationships from synchronization patterns. *Physical review. E, Statistical physics, plasmas, fluids, and related interdisciplinary topics* **61,** 5142-5148.

Quiroga, R. Q., Kreuz, T. and Grassberger, P. (2002) Event synchronization: a simple and fast method to measure synchronicity and time delay patterns. *Physical review. E, Statistical, nonlinear, and soft matter physics* **66,** 041904.

Righart, R., Biberacher, V., Jonkman, L. E., Klaver, R., Schmidt, P., Buck, D., Berthele, A., Kirschke, J. S., Zimmer, C., Hemmer, B., Geurts, J. J. G. and Muhlau, M. (2017) Cortical pathology in multiple sclerosis detected by the T1/T2-weighted ratio from routine magnetic resonance imaging. *Annals of neurology* **82,** 519-529.

Saenger, V. M., Kahan, J., Foltynie, T., Friston, K., Aziz, T. Z., Green, A. L., Van Hartevelt, T. J., Stevner, A. B. A., Fernandes, H. M., Mancini, L., Thornton, J., Yousry, T., Limousin, P., Zrinzo, L., Hariz, M., Kringelbach, M. L. and Deco, G. (2017) Uncovering the underlying mechanisms and whole-brain dynamics of therapeutic deep brain stimulation for Parkinson’s disease [bioRxiv 083162]. *Scientific Reports* **7,** 9882.

Schreiber, T. and Schmitz, A. (1996) Improved Surrogate Data for Nonlinearity Tests. *Physical review letters* **77,** 635-638.

Sengupta, S., Basak, S. and Peters, R. (2019) Particle Swarm Optimization: A Survey of Historical and Recent Developments with Hybridization Perspectives. *Machine Learning and Knowledge Extraction* **1,** 157-191.

Seth, A. K., Chorley, P. and Barnett, L. C. (2013) Granger causality analysis of fMRI BOLD signals is invariant to hemodynamic convolution but not downsampling. *NeuroImage* **65,** 540-555.

van den Heuvel, M. P. and Sporns, O. (2011) Rich-club organization of the human connectome. *The Journal of neuroscience : the official journal of the Society for Neuroscience* **31,** 15775-15786.

Vidaurre, D., Hunt, L. T., Quinn, A. J., Hunt, B. A. E., Brookes, M. J., Nobre, A. C. and Woolrich, M. W. (2018) Spontaneous cortical activity transiently organises into frequency specific phase-coupling networks. *Nat Commun* **9,** 2987.
Weber, I., Florin, E., von Papen, M. and Timmermann, L. (2017) The influence of filtering and downsampling on the estimation of transfer entropy. *PloS one* **12,** e0188210.
Zamora-Lopez, G., Zhou, C. and Kurths, J. (2010) Cortical hubs form a module for multisensory integration on top of the hierarchy of cortical networks. *Frontiers in neuroinformatics* **4,** 1.

**Decision Letter, first revision:**

11th August 2020

Dear Gus,

RE: "Revisiting the Global Workspace: Orchestration of the functional hierarchical organisation of the human brain"

Thank you for submitting your revised manuscript and for all your work on the revision. I am sorry it took me a little while to editorially check your revision.

Although you revised your manuscript in response to the reviewer comments, it does not fully comply with our editorial policies and formatting requirements.

In particular, there are multiple instances where statistics reporting does not meet our requirements, which include
a) naming the statistical test (e.g. for the comparisons shown in Figure 5, this should be stated in the Figure legend)
b) reporting full statistics, as explained in the checklist (attached) for statistical tests, including both parametric and non-parametric tests (this pertains for example to the linear correlations, reported in the main text and in Figure 2, but also the contrast mentioned under a).

Before we can send the manuscript back to our reviewers, we ask that you revise it to ensure that it complies fully with our policies and is formatted according to our requirements. I have attached another copy of our checklist, as well as the template document that exemplifies our formatting and policy requirements. If you are uncertain as to how to address any of the points in the checklist, please don't hesitate to contact me.

Please use the link below to submit your revised manuscript and related files:

***[REDACTED]***

<strong>Note:</strong> This URL links to your confidential home page and associated information about manuscripts you may have submitted, or that you are reviewing for us. If you wish to forward this email to co-authors, please delete the link to your homepage.

Thank you in advance for attending to these requests and I look forward to receiving your revised

manuscript.

Best wishes
Marike

Marike Schiffer, PhD
Senior Editor
Nature Human Behaviour

**Decision Letter, second revision:**

15th September 2020

*Please ensure you delete the link to your author homepage in this e-mail if you wish to forward it to your co-authors.

Dear Gus,

Thank you once again for submitting your revised manuscript, entitled "Revisiting the Global Workspace orchestrating the hierarchical organisation of the human brain," and for your patience during the re-review process.

Your manuscript has now been evaluated by our referees, and in the light of their advice I am delighted to say that we can in principle offer to publish it. First, however, we would like you to revise your paper to address the editorial concerns, and to ensure that it complies with our Guide to Authors at http://www.nature.com/nathumbehav/info/gta.

The primary editorial concern is presentational. We understand your enthusiasm for the project. Nonetheless, as a policy, we maintain that that the evaluation of a piece of work is best left to the reader and that any inclusion of statements that praise the nature and advance made by the authors compromises the credibility of the work. Please remove all statements that qualitatively evaluate your work throughout the manuscript. For instance, 'careful' should be dropped as a characterization of your design and 'state-of-the-art' should likewise be deleted as a characterization of your method. Statements such as the following (taken from the abstract and also reiterated in the main text) must be eliminated:

"Overall, this is a major step forward in understanding the complex choreography of the functional hierarchical organisation of the human brain."

Statements such as the following:

"This is strong causal evidence for the major significance of FRIC in orchestrating functional organisation."

and

"Importantly, as a further proof of causal significance of the core FRIC regions..."

must be rephrased to be matter-of-fact:

"This provides evidence for the role of FRIC in orchestrating functional organisation."

"To provide further evidence of causal significance of the core FRIC regions..."

Providing matter-of-fact descriptions, without any qualitative characterisations, must guide the final revisions to your manuscript.

Moreover, there are a fair number of repetitions of procedural aspects, characteristics of the data and approach, and procedural steps in the text, that compromise reader experience. We ask you to limit repetitions to the absolute minimum required to keep readers oriented towards what you did and what you found.

One of the main reasons for delays in formal acceptance is failure to fully comply with editorial policies and formatting requirements. To assist you with finalizing your manuscript for publication, I attach a checklist that lists all of our editorial policies and formatting requirements. I also attach a template document, which exemplifies our policies and formatting requirements.

Please attend to *every item* in the checklist and upload a copy of the completed checklist with your submission. I have highlighted in the checklist items that require your attention. I also mention here a few points that are frequently missed and can cause delays:

1) Ensure that all corresponding authors have linked their ORCID to their account on our online manuscript handling system. This is very frequently missed and invariably causes delays in formal acceptance.

2) Ensure that you provide all of the materials requested in the attached checklist and below with your final submission. Please note that the Licence to Publish needs to be hand-signed.

3) Given the nature of the project, we would strongly recommend openly sharing the code. Given the missing Code Availability Statement, I am not sure whether you have considered this. Please contact me if you have any questions.

Nature Human Behaviour offers a transparent peer review option for new original research manuscripts submitted from 1st December 2019. We encourage increased transparency in peer review by publishing the reviewer comments, author rebuttal letters and editorial decision letters if the authors agree. Such peer review material is made available as a supplementary peer review file. <b>Please state in the cover letter ‘I wish to participate in transparent peer review’ if you want to opt in, or ‘I do not wish to participate in transparent peer review’ if you don’t.</b> Failure to state your preference will result in delays in accepting your manuscript for publication.
Please note: we allow redactions to authors’ rebuttal and reviewer comments in the interest of confidentiality. If you are concerned about the release of confidential data, please let us know specifically what information you would like to have removed. Please note that we cannot incorporate redactions for any other reasons. Reviewer names will be published in the peer review files if the reviewer signed the comments to authors, or if reviewers explicitly agree to release their name. For

more information, please refer to our <a href="https://www.nature.com/documents/nr-transparent-peer-review.pdf" target="new">FAQ page</a>.

We hope to hear from you within 10 days; please let us know if the revision process is likely to take longer.

To submit your revised manuscript, you will need to provide the following:
• Cover letter
• Manuscript text (not including the figures) in .docx or .tex format
• Individual figure files (one figure per file)
• Extended Data & Supplementary Information, as instructed
• Reporting summary
• Editorial policy checklist
• License to publish
• Third-party rights table (if applicable)
• Suggestions for cover illustrations (if desired)

Consortia authorship:
For papers containing one or more consortia, all members of the consortium who contributed to the paper must be listed in the paper (i.e., print/online PDF). If necessary, individual authors can be listed in both the main author list and as a member of a consortium listed at the end of the paper. When submitting your revised manuscript via the online submission system, the consortium name should be entered as an author, together with the contact details of a nominated consortium representative. See https://www.nature.com/authors/policies/authorship.html for our authorship policy and https://www.nature.com/documents/nr-consortia-formatting.pdf for further consortia formatting guidelines, which should be adhered to prior to acceptance.

Reviewer Recognition:
In recognition of the time and expertise our reviewers provide to Nature Human Behaviour's editorial process, we would like to formally acknowledge their contribution to the external peer review of your manuscript entitled "Revisiting the Global Workspace orchestrating the hierarchical organisation of the human brain". For those reviewers who give their assent, we will be publishing their names alongside the published article.

Please use the following link for uploading these materials:
***[REDACTED]***

If you have any further questions, please feel free to contact me.

Best wishes,
Marike

Marike Schiffer, PhD
Senior Editor
Nature Human Behaviour

Reviewer #1:

Remarks to the Author:
No further concerns.

Reviewer #2:
Remarks to the Author:
The revised manuscript has addressed all my concerns and I have no further question. The quality of manuscript is improved very much and it is ready for publication.

Reviewer #3:
Remarks to the Author:
The authors have addressed my concerns

**Final Decision Letter:**

Dear Gus,

We are pleased to inform you that your Article "Revisiting the Global Workspace orchestrating the hierarchical organisation of the human brain", has now been accepted for publication in Nature Human Behaviour.

Before your manuscript is typeset, we will edit the text to ensure it is intelligible to our wide readership and conforms to house style. We look particularly carefully at the titles of all papers to ensure that they are relatively brief and understandable.

The subeditor may send you the edited text for your approval. Once your manuscript is typeset you will receive a link to your electronic proof via email within 20 working days, with a request to make any corrections within 48 hours. If you have queries at any point during the production process then please contact the production team at rjsproduction@springernature.com. Once your paper has been scheduled for online publication, the Nature press office will be in touch to confirm the details.

Acceptance of your manuscript is conditional on all authors' agreement with our publication policies (see http://www.nature.com/nathumbehav/info/gta). In particular your manuscript must not be published elsewhere and there must be no announcement of the work to any media outlet until the publication date (the day on which it is uploaded onto our web site).

The Author's Accepted Manuscript (the accepted version of the manuscript as submitted by the author) may only be posted 6 months after the paper is published, consistent with our <a href="http://www.nature.com/authors/policies/license.html">self-archiving embargo</a>. Please note that the Author’s Accepted Manuscript may not be released under a Creative Commons license. For Nature Research Terms of Reuse of archived manuscripts please see: <a href="http://www.nature.com/authors/policies/license.html#terms">http://www.nature.com/authors/policies/license.html#terms</a>

If you have posted a preprint on any preprint server, please ensure that the preprint details are updated with a publication reference, including the DOI and a URL to the published version of the article on the journal website.

An online order form for reprints of your paper is available at <a href="https://www.nature.com/reprints/author-reprints.html">https://www.nature.com/reprints/author-reprints.html</a>. All co-authors, authors' institutions and authors' funding agencies can order reprints using the form appropriate to their geographical region.

We welcome the submission of potential cover material (including a short caption of around 40 words) related to your manuscript; suggestions should be sent to Nature Human Behaviour as electronic files (the image should be 300 dpi at 210 x 297 mm in either TIFF or JPEG format). Please note that such pictures should be selected more for their aesthetic appeal than for their scientific content, and that colour images work better than black and white or grayscale images. Please do not try to design a cover with the Nature Human Behaviour logo etc., and please do not submit composites of images related to your work. I am sure you will understand that we cannot make any promise as to whether any of your suggestions might be selected for the cover of the journal.

You can now use a single sign-on for all your accounts, view the status of all your manuscript submissions and reviews, access usage statistics for your published articles and download a record of your refereeing activity for the Nature journals.

To assist our authors in disseminating their research to the broader community, our SharedIt initiative provides you with a unique shareable link that will allow anyone (with or without a subscription) to read the published article. Recipients of the link with a subscription will also be able to download and print the PDF.

As soon as your article is published, you will receive an automated email with your shareable link.

We look forward to publishing your paper.

Best wishes,
Marike

Marike Schiffer, PhD
Senior Editor
Nature Human Behaviour

P.S. Click on the following link if you would like to recommend Nature Human Behaviour to your librarian http://www.nature.com/subscriptions/recommend.html#forms

** Visit the Springer Nature Editorial and Publishing website at <a href="http://editorial-jobs.springernature.com?utm_source=ejP_NHumB_email&utm_medium=ejP_NHumB_email&utm_campaign=ejp_NHumB">www.springernature.com/editorial-and-publishing-jobs</a> for more information

about our career opportunities. If you have any questions please click <a href="mailto:editorial.publishing.jobs@springernature.com">here</a>.**
